# Fluid and Imaging Biomarkers in Huntington’s Disease

**DOI:** 10.1007/s11910-026-01522-1

**Published:** 2026-09-24

**Authors:** Elizabeth L. Broom, Joelle Hanson-Baiden, Lauren M. Byrne

**Affiliations:** Huntington’s Disease Centre, Department of Neurodegenerative Disease, UCL Queen Square Institute of Neurology, University College London, London, WC1B 5EH 2nd Floor Russell Square House, 10-12 Russell Square, UK

**Keywords:** Huntington’s disease, Biomarkers, Neurofilament light, Mutant huntingtin, Structural MRI, Regulatory qualification

## Abstract

**Purpose of Review:**

Growing interest in earlier and mechanism-targeted interventions for Huntington’s disease (HD) has brought focus on biomarkers capable of informing trial design and regulatory pathways. This review discusses recent advances in fluid and neuroimaging biomarkers since the introduction of the HD-Integrated Staging System (HD-ISS), focusing on candidates with the most relevant evidence for regulatory applications.

**Recent Findings:**

Neurofilament light (NfL) has become a versatile biomarker for prognostic enrichment and safety monitoring. Mutant huntingtin (mHTT) and somatic expansion ratio (SER) provide mechanistic evidence of target engagement. HD-YAS showed SER predicts striatal atrophy over multi-year intervals, but its short-term sensitivity remains uncertain. Structural MRI remains the most sensitive imaging marker of progression, whilst PET and diffusion imaging offer mechanistic insight but limited temporal sensitivity.

**Summary:**

The most advanced biomarkers now span neuronal injury, target engagement and neurodegeneration. Moving toward qualified endpoints will require harmonised methods, improved analytical validation and stronger evidence linking biomarker change to clinical benefit.

**Supplementary Information:**

The online version contains supplementary material available at https://doi.org/10.1007/s11910-026-01522-1.

## Introduction: Transformations in Therapeutic Development for Huntington’s disease

Huntington’s disease (HD) is an autosomal dominant neurodegenerative disorder characterised by psychiatric, cognitive and motor symptoms that typically emerge in mid-life [1]. It is caused by an expanded cytosine-adenosine-guanine (CAG) repeat tract in exon 1 of the huntingtin gene (*HTT*), discovered in 1993 [2]. Despite this genetic understanding, repeated clinical trial failures have left HD without any approved disease-modifying therapies (DMTs). Current therapies treat symptoms, providing only modest relief of motor or psychiatric disturbances. However, the last decade has heralded an expanding range of investigational therapies targeting the mechanisms underlying disease progression, bringing hope and complexity to the HD therapeutic landscape [3].

Among these, huntingtin (HTT)-lowering strategies have gained prominence as a rational, disease-specific approach to counteract the toxic effects of mutant HTT (mHTT). Current HTT-lowering approaches span the central dogma, ranging from DNA-directed transcriptional silencing [4]; through RNA-targeted modalities such as antisense oligonucleotides (ASOs), RNA interference (RNAi), splicing modulators, and microRNA gene therapies [5]; to protein-level interventions designed to enhance HTT clearance [3, 6]. RNA-targeted strategies have advanced furthest through the pipeline, with uniQure’s gene therapy AMT-130 and Novartis’s splicing modulator votoplam having recently met their Phase I/II and Phase II primary endpoints, respectively; both demonstrating HTT-lowering and early signs of clinical improvement [7, 8]. Allele-selective strategies are also being explored to preferentially suppress mHTT while preserving wild-type HTT (wtHTT), employing single nucleotide polymorphism (SNP)-targeting (WVE-003, Wave Life Sciences) and CAG repeat-directed (VO659, Vico Therapeutics) approaches [3].

Beyond HTT-lowering, genome-wide association studies (GWAS) have implicated DNA damage response (DDR; mainly mismatch repair, MMR) genes as modifiers of clinical symptom onset [9]. Mechanistic work suggests these modifiers influence the propensity for somatic CAG repeat expansion, now recognised as a key driver of progression, associated with the age of diagnosis and disease progression rates [10]. These insights have directed interest toward DDR modulation to stabilise repeat expansion.

**Fig. 1:**
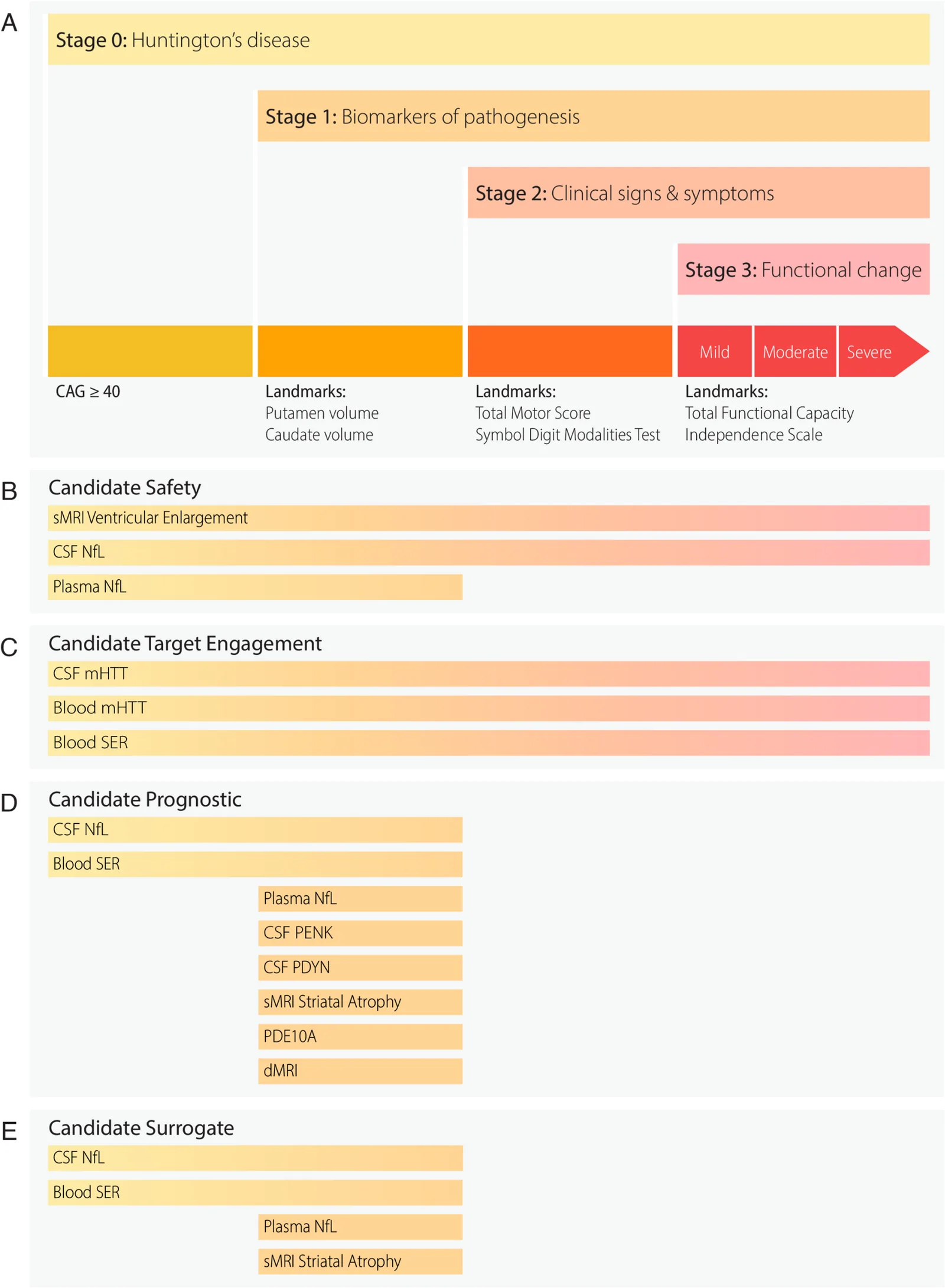
The Huntington’s Disease Integrated Staging System. (**A**) Overview of the HD-ISS. The Huntington’s Disease Integrated Staging System (HD-ISS) provides a clear framework for classifying persons with HD (pwHD) from birth through end-stage [11]. The HD-ISS comprises four stages: Stage 0 begins at birth with the inheritance of an expanded CAG tract in the huntingtin gene (CAG ≥ 40) but precedes any discernible signs of disease; Stage 1 is marked by striatal atrophy; Stage 2 by motor and cognitive impairment; and Stage 3 by functional decline. By incorporating key biological landmarks of early pathogenesis—namely caudate and putamen volume—the HD-ISS enables earlier disease classification, supporting intervention trials aimed at preventing progression in earlier stages. (**B**-**E**) Our perspective on key biomarker categories across HD-ISS stages based on the literature. Shown are the biomarker domains most relevant for qualification within each HD-ISS stage, along with leading candidates within each category to date. Highlighting stage-specific biomarker utility provides a structured framework for aligning biomarker development with disease biology and therapeutic objectives. (Fig. 1A adapted from The Lancet Neurology, Tabrizi et al., 2022;21:632–44 [11], with permission from Elsevier.)

Most HD trials to date have enrolled symptomatic persons with HD (pwHD), relying on clinical outcome assessments as endpoints [3]. However, repeated failures and decades-long pathogenic changes detectable via imaging and fluid biomarkers, suggest that interventions may need to occur earlier, before clinical symptoms emerge, to ensure the greatest potential for clinical benefit. In recognition of this, the Huntington’s Disease Integrated Staging System (HD-ISS) was introduced in 2022, which classifies pwHD from birth to end-stage using clinical and biological landmarks (Fig. 1) providing a framework for earlier disease classification and prevention trial design [11].

These shifts toward earlier and more mechanism-specific interventions have fundamentally changed the HD research landscape, amplifying the demand for sensitive biomarkers to guide patient selection, confirm target engagement, and measure treatment effects in the absence of overt clinical symptoms. Here, we performed a structured review of fluid and imaging biomarkers in HD since the introduction of the HD-ISS in 2022 (Supplementary Figs. 1 and 2), highlighting regulatory applications and opportunities for qualification and integration into multimodal strategies to support DMT development. Historical data preceding 2022 are summarised in the Supplementary Tables 3 and 4, and where evidence remains uncertain, we provide perspective and outline priorities for future research.

## Recent evidence in Biomarker Applications

**Fig. 2:**
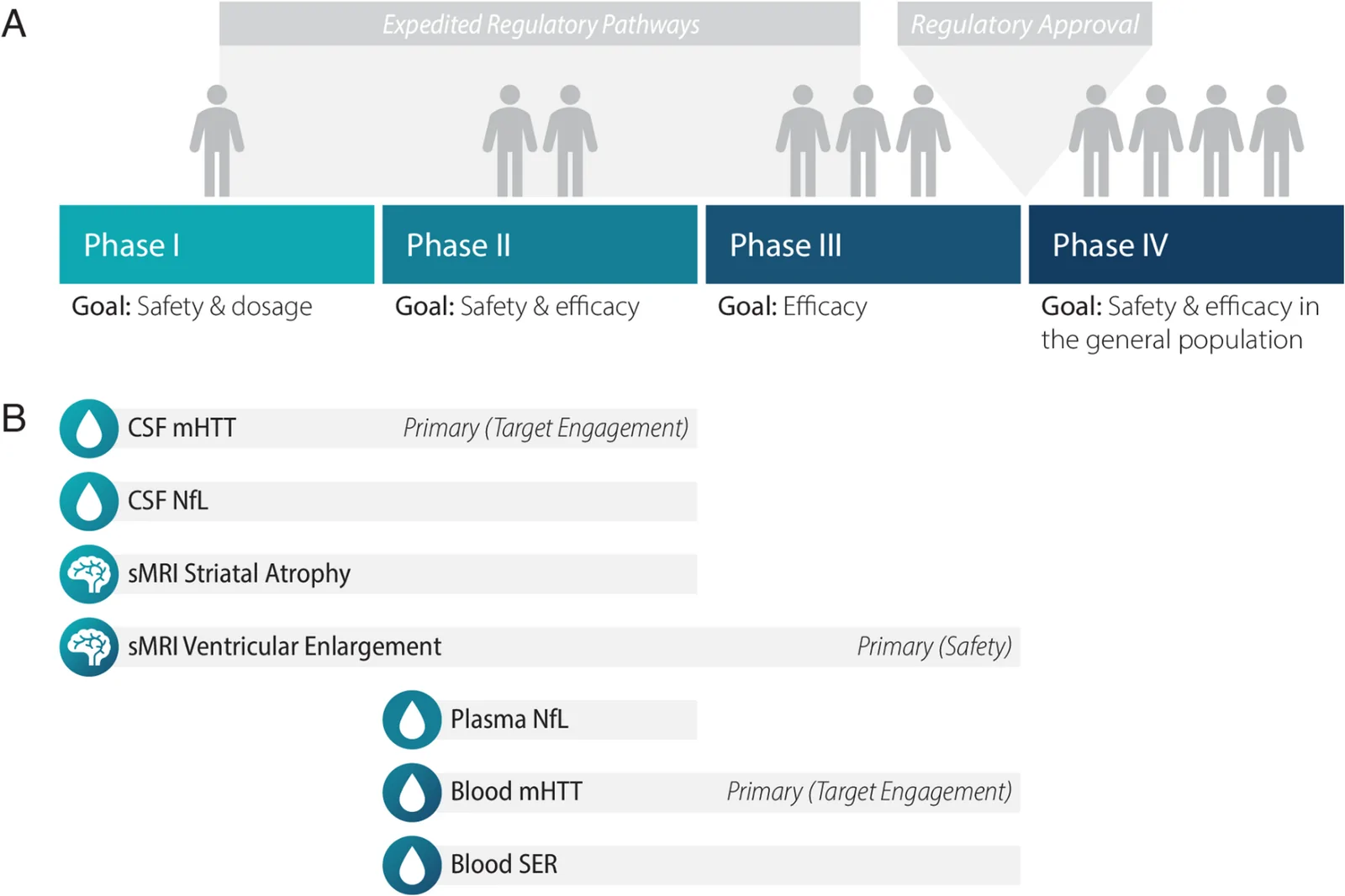
Application of fluid and imaging biomarkers across Huntington’s disease clinical trial phases. (**A**) Schematic illustrating the typical phases of drug development, the primary goals for each phase, and (**B**) current applications of the fluid and imaging biomarkers discussed in this review in ongoing clinical trials, as primary (mHTT, target engagement; sMRI ventricular enlargement, safety), secondary and exploratory endpoints. AB-1001, Phase I/II NCT05541627: CSF NfL, CSF mHTT; AMT-130, Phase I/II NCT04120493, NCT05243017: CSF NfL, CSF mHTT, sMRI; WVE-003, Phase I/II NCT05032196: CSF NfL, CSF mHTT, sMRI; Tominersen, Phase II NCT05686551: Plasma NfL, CSF NfL, CSF mHTT, sMRI; Votoplam, Phase II NCT05358717: CSF NfL, CSF mHTT, sMRI; SKY-0515, Phase II/III NCT06873334: Blood mHTT, blood SER. Abbreviations: mHTT, mutant huntingtin; PMS1, postmeiotic segregation increased 1; NfL, neurofilament light; sMRI, structural magnetic resonance imaging; SER, somatic expansion ratio

Biomarker qualification requires validation within a clearly defined context of use (COU) [12], specifying both its function and intended application (Table 1), including the target population, measurement approach and decision the biomarker is intended to support. In this section, we summarise recent advances in fluid and neuroimaging biomarkers, prioritising candidates with replicated evidence or use in interventional studies that show the greatest potential to accelerate HD therapeutic development. Each biomarker is discussed within its proposed category, together with the disease stage and position in the development pipeline for which it appears most suitable, to help inform the development of a future COU (Figs. 1 and 2). Additional exploratory candidates are summarised in Supplementary Tables 2 and 3 and classified according to their current level of evidence—exploratory, supported by replicated evidence or used in interventional studies.

**Table 1:** Definitions of regulatory terms used in this review, adapted from the (FDA)-National Institutes of Health (NIH) Biomarkers, EndpointS, and other Tools (BEST) glossary

**TOOLS**	
**Term**	**Definition**
Test, tool or instrument	The specific assay, imaging modality, device, or scale used to measure a biomarker or clinical outcome.
**BIOMARKERS**	
**Term**	**Definition**
Biomarker	A defined characteristic that is measured as an indicator of normal biological processes, pathogenic processes, or responses to an exposure or intervention.
Monitoring biomarker	Measured serially to assess disease status or response to an intervention over time.
Pharmacodynamic/response biomarker	Indicates a biological response has occurred following exposure to an intervention, reflecting target engagement or pathway modulation.
Predictive biomarker	Identifies individuals more likely to experience a favourable or unfavourable effect from a specific intervention.
Prognostic biomarker	Indicates the likelihood of a future clinical event, disease progression, or outcome independent of treatment.
Safety biomarker	Indicates the likelihood, presence, or extent of toxicity or adverse effects following exposure to an intervention.
**ENDPOINTS**	
**Term**	**Definition**
Clinical endpoint	A measure that directly assesses how a patient feels, functions, or survives, and is used to evaluate whether an observed change represents a clinically meaningful benefit, typically captured using a clinical outcome assessment (COA).
Biomarker-based endpoint (surrogate endpoint)	A biomarker used as a substitute for a clinical endpoint, intended to predict clinical benefit or harm. Depending on the strength of evidence supporting their ability to predict clinical benefit, they may be designated as *candidate*, *reasonably likely* or *validated* surrogate endpoints.

[12]

### Pharmacodynamic Biomarkers

Pharmacodynamic biomarkers assess the biological response to intervention, without necessarily establishing a link to clinical efficacy or disease outcomes. They are especially valuable in early-phase trials (Phase I and IIa), where the primary goals are to assess treatment safety, confirm target engagement and find an appropriate therapeutic dose [12].

#### Safety

Safety biomarkers provide early readouts of treatment-related risk (Phase I) and are essential across all trial phases.

##### Neurofilament Light

Neurofilament light protein (NfL) is a well-established marker of neuroaxonal injury that consistently correlates with HD severity in CSF and blood, as detailed in Supplementary Materials Table 4. For this reason, NfL has increasingly been included as an exploratory or secondary endpoint in HD clinical trials. However, recent observations of treatment-related NfL elevations have led to its recognition as an emerging safety biomarker.

A Phase III trial of the HTT-lowering ASO tominersen (NCT03761849) reported dose-dependent spikes in CSF NfL alongside increased levels of leukocytes and total CSF protein, consistent with an inflammatory response; these changes preceded adverse clinical events and contributed to the early termination of the trial [13, 14]. Similarly, in a trial of the small-molecule splicing modulator branaplam (NCT05111249) serum NfL elevations were detected after nine weeks of treatment, coinciding with peripheral neuropathy, leading to study termination; findings that have since been corroborated in vitro [15, 16].

Importantly, NfL signals must be interpreted in the context of the therapeutic modality and route of administration. For instance, in the Phase I/II trial of gene therapy AMT-130 (NCT04120493), transient acute elevations in CSF NfL were observed immediately following neurosurgical administration, but levels subsequently returned to baseline and even fell below, suggesting these spikes reflected procedural effects rather than drug-induced neurotoxicity [7, 17].

##### Magnetic Resonance Imaging

Magnetic resonance imaging (MRI) has provided key safety signals in HD trials as a result of adverse effects. Frequent high-dosing of tominersen was associated with ventricular enlargement exceeding natural history expectations [13]. Additionally, in gene therapy studies such as AMT-130, MRI supports perioperative planning and monitoring for oedema or haemorrhage [18].

#### Target Engagement

Target engagement biomarkers confirm that a therapy modulates its intended molecular target in vivo, providing proof-of-concept and informing dose optimisation in early-phase trials.

##### Huntingtin

With HTT-lowering remaining the leading intervention strategy in HD, both mutant and total HTT serve as key pharmacodynamic biomarkers. Since the non-allele-selective ASO tominersen became the first therapy to demonstrate mHTT-lowering in humans in 2017, six clinical candidates have successfully lowered CSF or plasma mHTT in trials, including four non-allele-selective and two allele-selective therapies [3].

Although widely employed in HTT-lowering trials, accurate quantification of mHTT in biofluids remains a major technical challenge due to its extremely low abundance, molecular heterogeneity, and variable polyglutamine (polyQ) repeat lengths and conformations [19]. Current quantification relies on ultrasensitive immunoassays that use antibody pairs to capture and detect HTT species, with the MW1 polyQ-targeted probe commonly employed for mHTT detection. However, MW1 exhibits preferential, rather than specific, binding to mHTT and is affected by polyQ length, complicating absolute quantification [19]. As a result, the field has shifted toward reporting relative rather than absolute concentrations, emphasising caution when interpreting historical data.

Despite advances in assay optimisation, the sensitivity of current CSF mHTT detection methods remains a significant challenge, particularly at the earliest disease stages where levels are extremely low. In the far-from-clinical motor diagnosis (CMD) HD Young Adult Study (HD-YAS) comprising 57 HD-ISS0-1 pwHD approximately 24 years prior to their predicted clinical symptom onset at base and 46 age-matched controls, CSF mHTT concentrations frequently fell below quantifiable levels, with only ~38% of samples exceeding the lower limit of quantification with an acceptable coefficient of variance below 30% [20]. Optimisation efforts have led to the independent validation of the single-molecule counting (SMC) assay, which now demonstrates improved batch consistency and signal-to-noise ratio—bringing mHTT quantification closer to clinical-grade reliability suitable for use as a regulatory endpoint [21, 22].

Parallel developments are expanding beyond CSF to peripheral biofluids, with promising detection of mHTT in tear fluid [23]—at levels approximately 230-fold higher than in CSF—and in saliva [24], where concentrations correlate with CAG repeat length and clinical severity. Ongoing innovation in isoform- and aggregation-specific assays continues to enhance understanding of HTT biology with potential to improve the precision of therapeutic monitoring [25, 26].

##### Somatic Expansion Rate

With the growing development of MMR-targeted therapies to reduce *HTT* CAG somatic expansion, accurate quantification of somatic expansion rates *in vivo* has become increasingly important for confirming target engagement and establishing proof-of-concept in trials. Numerous studies have demonstrated that somatic expansion occurs most prominently in striatal tissue [10, 27–29], but direct measurement in brain is not feasible in clinical trials, creating a need for sensitive methods to quantify expansion in accessible tissues such as blood. Traditional PCR-based fragment analysis of the somatic expansion ratio (SER)—the relative increase in somatic versus progenitor products—is limited by PCR bias and sensitivity, preventing detection of low-frequency, larger expansions in these tissues [30]. Recent advances in ultra-deep sequencing technologies have improved the sensitivity of SER measurement, supporting more robust quantification in peripheral DNA [31], revealing that increased blood-derived SER rates of change correlate with earlier clinical motor diagnosis (CMD), indicating blood SER is a proxy for brain expansion dynamics [32]. Although still exploratory, blood SER may prove particularly valuable in early-stage therapeutic development.

In the 4.5-year follow-up of the far-from-CMD HD-YAS cohort, longitudinal increases in blood-derived SER were detectable in HD-ISS0 pwHD via MiSeq sequencing [20]. The rate of change of SER predicted caudate and putamen atrophy, marking the first demonstration of blood SER associating with early-stage brain changes. Reflecting its translational potential, SER has been included as a secondary endpoint in a Phase II/III trial of a small-molecule splicing modulator (SKY-0515; NCT06873334), developed to lower HTT with reported secondary effects on the MMR protein postmeiotic segregation 1 (PMS1), a genetic modifier of CMD identified via GWAS [33, 34].

Whilst these technological advances support the quantification of SER in trials, human blood-based SER has been measured in only three studies via these methods [20, 32, 35]. Detectable changes typically occur over multi-year intervals, raising concerns about its practicality as a pharmacodynamic endpoint in short-duration trials. Defining the minimal timeframe and magnitude of change required for reliable detection will be essential to determine whether SER can serve as a feasible target engagement marker or if complementary biomarkers are needed.

### Prognostic Markers

Prognostic markers enable patient stratification and enrichment to identify individuals most likely to demonstrate measurable change within a trial. These are most relevant in Phase II and III trials where modelling disease trajectories helps to enrich cohorts and reduce required sample sizes. In addition, prognostic evidence lays the foundations for surrogate endpoints in future trials and eventually disease monitoring.

#### Neuronal Markers

##### Neurofilament Light

Since the seminal 2017 Track-HD retrospective analysis, which demonstrated that plasma NfL levels are elevated in pwHD, are CAG-dependent, and associate with both cross-sectional severity and longitudinal progression across numerous neuroimaging and clinical metrics, a substantial body of primary research spanning at least 20 independent cohorts have provided supporting evidence in human biofluid, as highlighted in Supplementary Table 4. Of these, 21 studies published since 2022 have examined NfL in CSF or blood across 14 HD cohorts (Supplementary Table 2).

Notably, in HD-YAS, CSF NfL was shown to rise more rapidly in pwHD than controls, from already elevated baseline levels [20]. Rates of increase in HD-ISS0 were comparable to those in HD-ISS1, despite caudate and putamen volume in HD-ISS0 remaining within the normal range, underscoring NfL’s potential as one of the earliest biomarkers of disease activity. Moreover, elevated baseline CSF and plasma NfL levels predicted subsequent atrophy across all reported brain regions, independent of age and CAG repeat length, with the strongest associations observed for caudate and putamen atrophy. Mean CSF NfL levels across both timepoints were also higher in pwHD who transitioned to HD-ISS1 compared to those who remained at HD-ISS0. By contrast, the rate of change in plasma NfL was not significant different between pwHD and controls, nor were baseline levels significantly higher in HD-ISS1 progressors, highlighting the superior sensitivity of CSF NfL in the far-from-CMD population.

Even so, evidence from the 14-year longitudinal Cambridge Huntington’s Sleep Study [36]—comprising 21 pwHD (6-55.9 years to CMD [ytCMD]) and 14 age-matched controls—demonstrated the strong prognostic utility of plasma NfL, with higher baseline levels and faster increases in pre-CMD, predicting more rapid decline in motor performance, executive function, and global cognition more than a decade later. Plasma NfL also showed excellent accuracy in discriminating pwHD who converted to.

Further evidence supporting NfL’s prognostic utility comes from a series of studies within an independent five-year longitudinal cohort of pwHD HD-ISS0-3 and healthy controls. Initial cross-sectional analyses of 26 pre-CMD, 39 post-CMD and 33 healthy controls confirmed that plasma NfL increases with advancing disease stage and correlates with predicted ytCMD [37]. Expanded analyses across two subcohorts (46 pre-CMD 66 post-CMD; 50 HD-ISS0, 64 HD-ISS1, 63 HD-ISS2, 63 HD-ISS3, 50 healthy controls) showed strong associations between plasma NfL, prognostic index (PIN) score, and predicted ytCMD, with levels below ~ 45 pg/mL identifying pwHD more than a decade from CMD, supporting stratification of HD-ISS1 by near-term progression risk [38, 39]. Longitudinal follow-up of 78 pwHD and 30 controls demonstrated progressive increases in plasma NfL across HD-ISS and PIN-derived stages—rising linearly in early disease and accelerating with progression—shifting from associations with cognition alone to both cognitive and motor decline in advances stages [40]. 

Collectively, these findings suggest that CSF and plasma NfL offer complementary value across the disease course, with CSF NfL capturing the earliest pathogenic changes and plasma NfL providing an accessible marker for prognostic enrichment near the HD-ISS2 transition. This accessibility is further supported by the recent validation of remote finger-prick sampling of capillary blood NfL as a reliable, minimally invasive alternative to venepuncture for NfL quantification [41].

#### Striatal Markers

Degeneration of striatal medium spiny neurons (MSNs) is a key hallmark of HD pathology [42]. MSNs represent around 95% of striatal neurons and form the major output of the basal ganglia, integrating dopaminergic and glutamatergic inputs through intracellular cyclic nucleotide signalling pathways [43]. Consequently, both CSF-based striatal proteins and molecular imaging measures reflecting MSN function have emerged as biomarker candidates for tracking disease progression.

##### Proenkephalin, Prodynorphin & Other Fluid Markers of Striatal Health

CSF neuropeptide precursors proenkephalin (PENK) and prodynorphin (PDYN), have been consistently reported lowered in pwHD compared to controls and associated with clinical severity (Supplementary Table 4; four papers since 2022 assessing either in human biofluids, Supplementary Table 2). In a mixed cohort of 47 post-CMD HD, 61 Parkinson’s disease (PD), 11 Alzheimer’s disease (AD), 14 sporadic amyotrophic lateral sclerosis (ALS) patients, and 92 healthy controls, both PENK- and PDYN-derived peptides were significantly lower in HD compared to all other groups, with correlations to clinical severity, highlighting this striatal specificity [44].

Further cross-sectional analyses of 26 CSF protein analytes from 8 pre-CMD, 16 post-CMD and eight age-matched controls [45], spanning markers of metabolism, immune function, neurotransmission and neuronal integrity—including NfL—found that PENK-derived peptides provided the strongest discriminatory power for distinguishing pwHD, and specifically pre-CMD, from healthy controls. By contrast, PDYN-derived peptides were most effective in differentiating early from late post-CMD, correlating strongly with clinical severity, while peptides from protein phosphatase 1 regulatory subunit 1B (PPP1R1B; also known as DARPP-32), best discriminated pre-CMD from early post-CMD. These analyses were performed using mass spectrometry, which is standard for peptidic markers like PENK and PDYN due to the need for high analytical specificity among multiple chemically similar peptide forms. Other markers, such as NfL, are typically quantified in clinical settings using ultrasensitive immunoassay, so direct comparison of biomarker performance to the more commonly cited immunoassay data should be interpreted cautiously.

In HD-YAS, reduced baseline CSF PENK levels predicted atrophy across all brain regions, while the rate of PENK decline—which was significantly greater in far-from-CMD pwHD compared to controls—further associated with the rates of caudate and putamen atrophy, independent of age and CAG repeat length [20]. These findings reinforce PENK’s utility as a sensitive marker of striatal integrity, capable of both stratifying early patient populations and predicting disease progression. Recent comparisons confirm that CSF PENK outperforms CSF NfL in discriminating HD-ISS Stage 0 from 1, predicting striatal-specific atrophy compared to the global white matter loss associated with NfL [46].

##### Phosphodiesterase-10 A Positron Emission Tomography

Phosphodiesterase-10 A (PDE10A) positron emission tomography (PET) provides a molecular readout of MSN integrity, as reductions in PDE10A binding reflect loss of intracellular cAMP and cGMP signalling and impaired dopaminergic-glutamatergic integration within the striatum [43]. Two studies pre-2022 showed reduced PDE10A binding up to ~ 25 years before motor onset, inversely correlating with CAG length, disease burden and motor and cognitive performance [47, 48], and declining progressively through pre-CMD and early post-CMD paralleling striatal atrophy and motor deterioration [48]. Building on this, the ongoing iMarkHD study is exploring PDE10A together with cannabinoid 1, histamine 2 and serotonin 2A PET ligands and multimodal MRI with longitudinal imaging over two years [49]. Interim analyses are expected to determine whether these molecular PET markers have sufficient sensitivity to support stratification in early-stage trials.

##### Structural Magnetic Resonance Imaging

Structural MRI (sMRI) measures, particularly striatal volume, are among the best characterised biomarkers of HD progression. Its inclusion in the HD-ISS as thresholds for transition from Stage 0 to 1 reflects the decades of research showing that the striatum undergoes progressive atrophy detectable well before motor onset (Supplementary Table 5). Recent studies confirm its sensitivity in early disease.

HD-YAS 4.5-year longitudinal analysis showed significantly greater annualised atrophy in both the caudate and putamen in pwHD participants compared to controls, as well as whole-brain, grey matter and white matter loss and accelerated ventricular expansion [20]. 23% of pwHD transitioned from HD-ISS0-1 over 4.5 years, demonstrating that volumetric sMRI is sensitive enough to capture measurable progression decades before motor onset [20].

sMRI also outperforms other imaging modalities for early progression tracking. A two-year study by Delva et al. (2023) assessed volumetric MRI and PET measures in parallel in pre-CMD and early post-CMD compared to controls. Annual caudate and putamen atrophy rates (4.5% in the caudate, 3.6% in the putamen) exceeded the sensitivity of concurrent PET measures over the same time period and correlated closely with decline in motor scores [50].

Beyond raw atrophy rates, sMRI data can be integrated into modelling approaches to improve prognostic enrichment and trial design, where participant recruitment and stratification traditionally rely on CAG length, cognitive performance and motor scores; placing less emphasis on in vivo imaging markers that more directly reflect underlying pathology years before diagnosis [51]. Barrett et al. (2024) integrated striatal volumes with age and CAG length across 630 pwHD from previously published cohorts PREDICT-HD, TRACK-HD, TrackON-HD and IMAGE-HD to develop predictive models for intrastriatal intervention screening [51]. Their logistic model predicted eligibility for volumetric surgical thresholds (putamen volume more than 5cm^3^) with an area under the curve of 0.92, correctly identifying 87% of eligible candidates whilst potentially reducing screening MRIs by nearly 40% [51]. This approach has direct relevance for intrastriatal gene-therapy trials such as AMT-130, offering a practical method to refine participant selection and enhance trial efficiency.

Using the same cohorts, Ghofrani-Jahromi et al. (2024) stratified patients based on data from 451 pwHD using machine-learning models trained on their baseline MRI, genetic and clinical features [52]. Their model predicted three-year ventricular enlargement with 24% greater accuracy than clinical measures alone and stratified patients into ‘fast’ (those with accelerated atrophy) and ‘slow’ progressors with ~ 81% accuracy [52].

Also recognising the individual variability in HD progression, Giannoula et al. (2025) used a dynamic time warping (DTW)-based unsupervised clustering approach on six years of longitudinal imaging and clinical data from 47 pwHD (22 pre-CMD and 25 post-CMD) to compare disease trajectories. Each participant’s trajectory was defined by caudate, putamen and nucleus accumbens volumes alongside Unified Huntington’s Disease Rating Scale total cognitive score, UHDRS_cogscore; total motor score, UHDRS-TMS; and the short-Problem Behaviour Assessment, PBA-s. This approach identified three distinct progression clusters: ‘slow progressors’ with relatively preserved striatal volumes and stable clinical function; ‘intermediate progressors’ with moderate rates of decline; and ‘fast progressors’ who had nearly double the rate of striatal atrophy compared to ‘slow progressors’ and more rapid motor and cognitive worsening [53]. These findings show integrating sMRI measures into clinical models utilising machine-learning enhances prognostic precision and supports enrichment strategies to identify participants most likely to show measurable progression in trials.

#### Complementary and Exploratory Biomarkers

A wider range of fluid and imaging candidates offer complementary mechanistic insight into HD pathology, though most currently lack the cohort sizes, replication or trial-endpoint use associated with markers such as NfL, mHTT, SER and sMRI. Further exploratory candidates are also summarised in Supplementary Tables 2 and 3.

##### Diffusion Magnetic Resonance Imaging

Diffusion magnetic resonance imaging (dMRI) allows interrogation of microstructural brain changes and is often used as an indicator of white matter integrity [54]. Yin et al. (2024) applied diffusion tensor imaging (DTI) and diffusion kurtosis imaging (DKI) in 22 pre-CMD and early post-CMD individuals [55]. Compared to 14 controls, pwHD showed widespread reductions in fractional anisotropy (FA) and even more extensive changes in mean kurtosis (MK), indicating deterioration of white matter tracts as a result of disease [55]. FA reflects the directional coherence of white matter tracts, with reductions indicating disrupted axonal integrity, whilst MK captures microstructural complexity with reductions reflecting loss of tissue organisation. Significant differences in MK were detected in 22 more brain regions than FA, and values in key regions were strongly inversely correlated with disease burden (CAP score used, *r* = -0.75). Combining DTI and DKI metrics further distinguished early-stage HD from controls with high accuracy (Youden’s index = -0.79, a composite measure of sensitivity and specificity) [55]. However, limitations of conventional DTI in HD are that FA reductions are non-specific, as they can reflect a mixture of demyelination, axonal loss or crossing-fibre effects. DKI thus provides added value by capturing tissue complexity inaccessible with standard DTI, which may account for its greater sensitivity in pre-CMD [55]. Both approaches require larger longitudinal studies to determine whether diffusion-derived measures reliably track the disease course over time and predict clinical milestones. In contrast, sMRI’s evidence base draws on larger, pooled, longitudinal and unlike diffusion metrics, striatal atrophy has been adopted as a trial endpoint (Fig. 2B), emphasising dMRI’s use as a mechanistic complement.

##### Glucose Metabolism and Synaptic Density Positron Emission Tomography

PET has been applied in observational studies to visualise metabolic and synaptic dysfunction. ^18^F-FDG PET, a marker of regional glucose metabolism, has demonstrated hypometabolism in the striatum and associative cortices which correlates with both motor and cognitive impairment [50]. However, in Delva et al. (2023)’s two-year study, metabolic decline did not reach statistical significance after correction [50]. In the same cohort, ^11^C-UCB-J PET, which binds to synaptic vesicle protein 2 A targeting synaptic density, revealed significantly reduced binding in the striatum in pre-CMD compared to controls, with further longitudinal decline extending into the frontal and parietal cortices, indicating a progression from subcortical to cortical synaptic degradation over two years [50]. These findings are consistent with the known early synaptic dysfunction in HD, previously reviewed by Ross and Tabrizi (2011) [56], however, the quantitative rate of decline in binding was small (~ 6% in the putamen over two years), limiting sensitivity for short-duration trials [50]. This is consistent with Delva et al.‘s (2023) direct comparison in the same cohort, where atrophy rates exceeded the sensitivity of concurrent PET measures over the same period [50], reinforcing that, despite its mechanistic value, PET has not matched sMRI’s sensitivity for tracking progression within trial-relevant timeframes.

## State of the Field After HD-ISS

Since the introduction of the HD-ISS [11], biomarker research has shifted toward earlier disease stages, with NfL and SER emerging alongside mHTT as leading fluid markers, and sMRI as the leading imaging measure. NfL offers broad utility—rising early, tracking severity, and responding to biological perturbations [7, 20, 38–40]—while mHTT and SER provide mechanistic evidence of target engagement [3, 20]. Imaging complements these measures: sMRI captures cumulative neurodegeneration [20–51], whereas PET and diffusion imaging offer mechanistic insights but limited short-term sensitivity [54–56]. Collectively, these findings support multimodal strategies combining responsive markers (NfL) with mechanistic and anatomical measures (mHTT, SER, sMRI) to optimise trial design across HD-ISS stages.

In our view, technical limitations remain a major barrier to wider adoption, and addressing these should be the field’s highest priority. Fluid biomarkers continue to face issues related to low abundance, molecular heterogeneity, and the absence of certified reference materials. This is particularly evident for mHTT assays, where polyQ-dependent antibody binding affects analytical accuracy and lower limits of quantification frequently fall below measurable levels in early-stage populations, as highlighted in HD-YAS [19, 20]. Striatal markers such as PENK and PDYN require mass spectrometry workflows that currently limit scalability [20, 44, 45]. In parallel, imaging biomarkers require site certification and centralised QC to mitigate hardware and processing variability. We consider these limitations a critical bottleneck; without improvements in sensitivity and standardisation, clinical-grade reliability will remain out of reach. We recommend advancing toward IVD-grade assay validation and regulatory-aligned imaging frameworks for further clinical implementation [21, 22].

Despite extensive research, no biomarker has been uniformly profiled across HD-ISS0–3. This partly reflects the small, single-cohort nature of much of the evidence base. Several findings summarised in Supplementary Table 3 are from cohorts with fewer than 100 participants and have not been independently replicated, alongside the predominance of cross-sectional designs that limit inference about individual trajectories over time. Given the infeasibility of assembling a single longitudinal cohort spanning the entire HD-ISS continuum, progress requires harmonised datasets before regulatory qualification can be considered. At present, variability in analytical platforms and scanners, calibration procedures, and data processing introduce batch effects that obscure biological signals and impede the development of regulatory-grade evidence. From our perspective, community-wide standardisation is now the rate-limiting step and should be prioritised over incremental biomarker discovery. Planned initiatives such as ENROLL-HD 2.0, with stage-tailored assessments, may help address these gaps, but also raises ethical and psychosocial considerations around pre-CMD staging disclosure [57].

Even with robust technical validation, interpretation of biomarker changes remains complex, requiring careful contextual consideration of therapeutic modality and timing. For instance, NfL elevations can reflect neurotoxicity or inflammation [13–16], yet acute peri-procedural spikes observed after neurosurgical gene therapy appear to be non-pathological [7, 17]. Similarly, sMRI excels for progression tracking but is less responsive to short-term effects, while PET offers mechanistic specificity but limited longitudinal sensitivity [20, 50]. We believe these nuances underscore the necessity of multimodal interpretation plans for accurate decision-making in both trials and regulatory contexts, particularly where evidence comes from cross-sectional comparisons alone.

## A Path to Surrogacy

In our view, the absence of validated surrogates is the single greatest obstacle to early intervention trials. Primary endpoints in HD trials remain limited to HD-ISS2 and 3, restricting evaluation of therapies in early disease. Surrogate biomarkers—those capable of predicting clinical benefit before measurable clinical changes occur—offer a path to earlier intervention. Under the FDA’s BEST framework, *validated* surrogates require direct evidence that biomarker change reliably predicts clinical benefit, whereas *reasonably likely* surrogates may support accelerated approval when supported by sufficient mechanistic and clinical rationale. Although no surrogate biomarkers are yet validated in HD, several *candidates* are emerging from the presented findings, though confirmatory evidence linking biomarker change to clinical benefit is still required.

NfL currently has the strongest evidence base for a future *reasonably likely* surrogate endpoint. Recent semi-mechanistic modelling supports this, establishing CSF NfL as a dynamic marker of the ongoing rate of neurodegeneration, rather than cumulative volume loss [58]. In AMT-130, high-dose patients showed CSF NfL levels below baseline over 36 months, contrasting the 30–40% expected rise with natural progression over three years [40], and showed 75% slowing of cUHDRS decline [7]. NfL also behaves reliably as a treatment-responsive biomarker in ALS, spinal muscular atrophy (SMA) and multiple sclerosis (MS), where NfL reductions accompany effective neuroprotective treatments [59–61], including its recent quantification in plasma alongside clinical outcomes to support the accelerated FDA approval of the ASO tofersen in SOD1-mediated ALS [59].

sMRI has been used as a secondary endpoint, a trial-specific outcome that provides supportive or exploratory information beyond the primary endpoint, in several interventional programmes. In the Phase Ib/IIa trial of WVE-003 (NCT05032196), greater CSF mHTT lowering correlated with slower caudate atrophy, which predicted less TMS worsening [62]. This association between pharmacodynamic response and structural preservation suggests caudate atrophy could serve as an imaging correlate of treatment response in HTT-lowering trials. The FDA has indicated that slowing of MRI-derived atrophy, together with evidence of mHTT lowering, could qualify under the *reasonably likely* surrogate framework [63].

## Endpoints for Prevention Trials

The discovery that somatic expansion processes begin in HD-ISS0 suggest interventions targeting MMR may need to be deployed far earlier than current trial designs allow [20]. This raises a critical question: how can we measure therapeutic benefit decades before CMD?

HD-YAS demonstrated that blood-derived SER increases were detectable in HD-ISS0 and predicted subsequent striatal atrophy over 4.5-years—the first evidence of peripheral SER reflecting early-stage brain changes [20]. Preclinical studies further support its mechanistic relevance, with splicing modulators such as branaplam and risidiplam slowing SER in HD cell models via PMS1-lowering [64]. Whether SER can provide a meaningful readout within early-phase trial timeframes remains unclear. Current evidence comes from multi-year observations, and its short-term sensitivity in HD-ISS0–1 is unknown, highlighting the need for longer follow-up or complementary biomarkers.

NfL could provide near-term sensitivity to neuronal injury and treatment effects, as sample size modelling in HD-YAS suggests NfL may outperform volumetric MRI for detecting change in HD-ISS0–1 [20]. We anticipate that prevention trials will require composite endpoints integrating mechanistic (SER), responsive (NfL), and structural biomarkers, as reliance on any single measure is unlikely to capture therapeutic benefit decades before CMD.

## Limitations

Some trial observations cited here derive from sponsor communications pending peer review [7, 8, 14, 16, 17]. These data should be interpreted cautiously until peer-reviewed results are available. We focused on biomarkers closest to regulatory applications; emerging candidates not reflected in this main text are summarised in Supplementary Tables 2 and 3.

## Conclusions

In conclusion, NfL, mHTT, SER and sMRI stand out as the most promising biomarkers for HD, offering complementary roles across responsiveness, mechanistic insight, and anatomical specificity. To realise their full potential, the field must prioritise harmonisation of protocols, rigorous analytical validation, and clear definition of context-of-use for regulatory pathways. Integrated biomarker strategies—combining fluid and imaging measures—will be critical for enabling earlier interventions, supporting accelerated approval frameworks, and powering prevention-stage trials. Ultimately, these efforts will transform HD therapeutic development by shifting the focus from symptomatic treatment to true disease modification.

## Key References

- Scahill RI, Farag M, Murphy MJ, Hobbs NZ, Leocadi M, Langley C, et al. Somatic CAG repeat expansion in blood associates with biomarkers of neurodegeneration in Huntington’s disease decades before clinical motor diagnosis. Nat Med. 2025;31:807–18. https://doi.org/10.1038/S41591-024-03424-6
  - Demonstrated that longitudinal increases in blood-derived somatic expansion predict striatal atrophy in far-from-CMD.
- Voysey ZJ, Owen NE, Holbrook JA, Malpetti M, Le Draoulec C, Spindler LRB, et al. A 14-year longitudinal study of neurofilament light chain dynamics in premanifest and transitional Huntington’s disease. J Neurol. Springer Science and Business Media Deutschland GmbH; 2024;271:7572–82. https://doi.org/10.1007/s00415-024-12700-x
  - Long-term evidence that plasma NfL predicts future motor and cognitive decline and accurately discriminates individuals who later convert to clinical motor onset.
- Barrett MJ, Negida A, Mukhopadhyay N, Kim JK, Nawaz H, Jose J, et al. Optimizing Screening for Intrastriatal Interventions in Huntington’s Disease Using Predictive Models. Movement Disorders. 2024;39:855–62. https://doi.org/10.1002/mds.29749
  - Showed that striatal volumetric MRI combined with age and CAG repeat length accurately predicts eligibility for intrastriatal interventions.
- Ghofrani-Jahromi M, Poudel GR, Razi A, Abeyasinghe PM, Paulsen JS, Tabrizi SJ, et al. Prognostic enrichment for early-stage Huntington’s disease: An explainable machine learning approach for clinical trial. Neuroimage Clin. 2024;43:103650.https://doi.org/10.1016/J.NICL.2024.103650
  - Demonstrated that MRI-based machine-learning models outperform clinical measures alone for predicting short-term neurodegeneration and stratifying progression rates.

## Electronic Supplementary Material

Below is the link to the electronic supplementary material.

Supplementary Material 1

## Data Availability

No datasets were generated or analysed during the current study.
