# Supplementary material for: Fluid and Imaging Biomarkers in Huntington’s Disease
Source: Curr Neurol Neurosci Rep. 2026 Sep 24;26(1):51. doi: 10.1007/s11910-026-01522-1 (PMC13612603; doi:10.1007/s11910-026-01522-1)
Supplement: Supplementary file 1 [file 11910_2026_1522_MOESM1_ESM.docx]

Supplementary Materials

**Table of Contents**

[Supplementary Methods	2](#_Toc240963333)

[Historical evidence of fluid and imaging biomarkers in Huntington’s disease	21](#_Toc240963334)

[Interventional Trials in Huntington’s Disease	25](#_Toc240963335)

[References	27](#_Toc240963336)

Supplementary Methods

Structured literature searches were conducted to identify primary studies published between 1^st^ January 2022 and 6^th^ October 2025 that investigate fluid and imaging biomarkers in Huntington’s disease (HD). Independent searches were performed separately for imaging and fluid biomarkers in PubMed.

The search strategies for both combined controlled vocabulary and free-text terms related to HD and biomarker terminology, as described in Supplementary Table 1.

**Supplementary Table 1.** Fluid and imaging search strategies for this review

| **Fluid** | |
| --- | --- |
| Terms: | ("huntington disease"[MeSH Terms] OR huntington*[tw]) AND (biofluid*[tw] OR fluid*[tw] OR "cerebrospinal fluid"[MeSH Terms] OR cerebrospinal fluid*[tw] OR csf[tw] OR "blood"[MeSH Terms] OR blood*[tw] OR "plasma"[MeSH Terms] OR plasma*[tw] OR "serum"[MeSH Terms] OR serum*[tw] OR "saliva"[MeSH Terms] OR saliva*[tw] OR "urine"[MeSH Terms] OR urine*[tw] OR "tears"[MeSH Terms] OR "tear"[tw] OR "tears"[tw] OR "sweat"[MeSH Terms] OR sweat*[tw] OR "extracellular vesicles"[MeSH Terms] OR extracellular vesic*[tw] OR "exosomes"[MeSH Terms] OR exosom*[tw]) AND ("biomarkers"[MeSH Terms] OR biomarker*[tw] OR marker*[tw]) |
| **Imaging** | |
| Terms: | ("huntington disease"[MeSH Terms] OR "huntington*"[tw]) AND ("biomarkers"[MeSH Terms] OR biomarker*[tw] OR marker*[tw] OR "neuroimaging"[MeSH Terms] OR imaging[tw]) AND ("multimodal imaging"[MeSH Terms] OR multimodal[tw] OR mri[tw] OR "magnetic resonance imaging"[MeSH Terms] OR fMRI[tw] OR "functional magnetic resonance imaging"[tw] OR "structural magnetic resonance imaging"[tw] OR rsfMRI[tw] OR "resting state fMRI"[tw] OR "resting state functional magnetic resonance imaging"[tw] OR "quantitative MRI"[tw] OR "quantitative magnetic resonance imaging"[tw] OR "MRS"[tw] OR "magnetic resonance spectroscopy"[MeSH Terms] OR "magnetic resonance spectroscopy"[tw] OR "diffusion tensor imaging"[MeSH Terms] OR DTI[tw] OR DWI[tw] OR "diffusion weighted imaging"[tw] OR "positron emission tomography"[MeSH Terms] OR "positron emission tomography"[tw] OR PET[tw] OR "tomography, emission computed, single photon"[MeSH Terms] OR SPECT[tw] OR QSM[tw] OR "quantitative susceptibility mapping"[tw] OR SWI[tw] OR "susceptibility weighted imaging"[tw] OR ASL[tw] OR "arterial spin labelling"[tw] OR "arterial spin labeling"[tw] OR "voxel based morphometry"[tw] OR VBM[tw] OR "surface based morphometry"[tw] OR SBM[tw] OR morphometry[tw] OR DCM[tw] OR "dynamic causal modelling"[tw] OR "dynamic causal modeling"[tw] OR volumetric*[tw] OR "brain cortical thickness"[MeSH Terms] OR "cortical thickness"[tw] OR "cerebral cortical thinning"[MeSH Terms] OR atrophy[MeSH Terms] OR atrophy[tw] OR "white matter"[MeSH Terms] OR "white matter"[tw] OR "grey matter"[tw] OR "gray matter"[MeSH Terms] OR "gray matter"[tw] OR caudate[tw] OR putamen[MeSH Terms] OR putamen[tw] OR neostriatum[MeSH Terms] OR neostriatum[tw] OR "corpus striatum"[MeSH Terms] OR "corpus striatum"[tw] OR striatum*[tw] OR striatal[tw] OR "cerebral cortex"[MeSH Terms] OR "cerebral cortex"[tw] OR thalamus[MeSH Terms] OR thalamus[tw] OR "thalamic nuclei"[MeSH Terms] OR "thalamic nuclei"[tw] OR thalamic[tw] OR tractography[tw] OR "functional connectivity"[tw] OR "machine learning"[MeSH Terms] OR "machine learning"[tw] OR "deep learning"[MeSH Terms] OR "deep learning"[tw] OR "radiomics"[MeSH Terms] OR "radiomics"[tw] OR "signal intensity"[tw] OR signal[tw] OR cross-sectional[tw] OR longitudinal[tw] OR longitudinally[tw]) |

Both searches were restricted to peer-reviewed primary studies written in English and involving only human participants. Pre-prints, meta-analyses and reviews were excluded. Eligibility was determined for through two-step screening by authors ELB and JHB separately for fluid and imaging searches. All search results were first imported into Rayyan [1] for duplicate removal and title and abstract screening, then the full-text of potentially relevant records were screened manually. Eligible references included both healthy controls and pwHD across the disease spectrum (excluding persons with Juvenile-onset HD), as well as empirical data on biomarker performance or relevance to disease mechanisms, progression or therapeutic development. Both cross-sectional and longitudinal designs were included, provided they offered mechanistic or clinical insight into respective biomarkers. Cross-sectional studies were included if they reported comparisons across disease stages or stratified participant groups (e.g. controls *vs* pwHD, pre-clinical motor diagnosis (CMD) *vs* post-CMD or HD-ISS staging).

Drug development studies were included only when biomarker data contributed to understanding disease biology, target engagement or treatment response, rather than solely pharmacokinetics. Studies were prioritised for inclusion if they contributed biomarker value within the FDA BEST framework, comprising pharmacodynamic, safety, prognostic and monitoring contexts of use. This allowed a consistent regulatory and translational framing of the evidence base across both fluid and imaging biomarkers. Conflicts were resolved through discussion with author LMB and consensus.

Press releases and other publicly available sponsor communications were reviewed to contextualise the chronology of biomarker use in clinical trials; however, as these sources are not peer reviewed, any observations derived from them should be interpreted cautiously and are acknowledged as a methodological limitation.

Extracted data from the final references included study design, sample size, participant demographics (e.g., disease stage, age, sex, cytosine-adenine-guanine (CAG) repeat length and years to motor onset (YTO), where available), biomarker modality and measurement method, and key quantitative outcomes, such as correlations with clinical measures, longitudinal change or treatment effects.

The fluid search identified 197 articles, of which 14 were included in the synthesis of the main text review, as detailed in Supplementary Figure 1. The imaging search identified 2681 articles, of which seven were included in the synthesis of the main text of the review, as detailed in Supplementary Figure 2. All main findings from the fluid and imaging searches are summarised in Supplementary Table 2 and Supplementary Table 3, respectively.

**n=197**

Records identified through PubMed search.

**n=164**

Records after removing duplicates and title screening.

**n=49**

Records after abstract screening.

**n=41**

Records that met final eligibility criteria following full-text screening. Of these, **n=14** studies were discussed in the main text.

Identification

Record screening

**n=8**

Records excluded at full-text screen:

- No empirical biomarker data

**Supplementary Figure 1.** The fluid biomarker search between 1st January 2022 and 6th October 2025. Out of 41 studies that met final inclusion criteria, 14 were discussed in the main text. Studies were prioritised for use in review synthesis if they contributed biomarker value within the FDA BEST framework.

**Supplementary Figure 2.** The imaging biomarker search between 1st January 2022 and 6th October 2025. Out of 28 studies that met final inclusion criteria, 7 were discussed in the main text of the review as they contributed biomarker value within the FDA BEST framework. Key findings of the remaining 21 studies are summarised in Supplementary Table 3.

**n=2681**

Records identified through PubMed search.

**n=348**

Records after removing duplicates and title screening.

**n=59**

Records after abstract screening.

**n=28**

Records that met final eligibility criteria following full-text screening. Of these, **n=7** studies were discussed in the main text.

Identification

Record screening

**n=31**

Records excluded at full-text screen:

- No empirical biomarker data (n=18)
- No link to disease mechanism or progression (n=11)
- Juvenile-onset HD only (n=2)

**Supplementary Table 2**. Main study findings of fluid biomarkers in Huntington’s disease from search between 1st January 2022 and 6th October 2025. Biomarker status indicates the current level of evidence and translational development for each candidate within the specified fluid and analytical technique. Exploratory biomarkers are supported by early or limited evidence. Replicated biomarkers have evidence across more than one independent cohort, study or analytical context. Biomarkers used in interventional studies have been incorporated into clinical trials as either exploratory or secondary endpoints. Currently no biomarkers are approaching regulatory qualification for a defined context of use.

| **First author (Year)** | **Analyte** | **Fluid** | **N** | **Aim** | **Key findings** | **Biomarker relevance** | **Biomarker status** |
| --- | --- | --- | --- | --- | --- | --- | --- |
| **Sampedro *et al.* (2022) [2]** | TDP-43 | Plasma | 36 pwHD | Investigate the clinical and structural brain correlates of plasma TDP-43 levels in HD. | Higher levels correlated with apathy severity, but not motor or general cognitive scores, as well as cortical thinning and increased microstructural diffusivity independent of mHTT and Tau levels. | Identifies plasma TDP-43 as a specific marker for apathy and frontotemporal degeneration in HD. | Exploratory |
| **Parkin *et al.* (2022) [3]** | NfL | Plasma | 46 pre-CMD, 66 post-CMD | Determine whether plasma NfL associates with the normalised PIN score and PIN-derived predicted years to motor onset (YTO) in HD. | Plasma NfL correlated with PIN scores and PIN-YTO in pre-CMD (not post-CMD), after adjustment for age and CAG. A cut-off of <45.01 pg/mL distinguished pre-CMD >10 years from predicted CMD *vs* ≤10 years. | Supports plasma NfL as a candidate marker for pre-CMD stratification; establishes a specific cut-off to enrich clinical trials with patients nearing onset. | Used in interventional studies |
| **Byrne *et al.* (2022) [4]** | NfL | Plasma | 30 pre-CMD, 9 JHD, 61 controls | Quantify plasma NfL in paediatric JHD and pre-CMD to assess utility for disease monitoring and clinical trials. | Plasma NfL significantly elevated in JOHD (~7-fold higher) and pre-CMD children <20 predicted YTO. Levels correlated with caudate/putamen atrophy. A cut-off of 9.64 pg/mL distinguished JOHD from controls (AUC 0.96). | Supports utility of plasma NfL for paediatric HD clinical trials. | Used in interventional studies |
| **Hellem *et al.* (2022) [5]** | Oxytocin | CSF, plasma | 69 post-CMD, 44 pre-CMD, 33 controls | Measure hypothalamic neuropeptide oxytocin levels in CSF and plasma and assess correlations with disease progression and social cognition. | CSF (but not plasma) oxytocin was significantly reduced in pwHD *vs* controls, specifically in post-CMD and those with cognitive impairment. Levels correlated with social cognition scores but not motor or disease burden scores. | Identifies CSF oxytocin as a biomarker for social cognitive impairment. | Exploratory |
| **Barschke *et al.* (2022) [6]** | PENK, PDYN | CSF | 47 post-CMD, 61 PD, 11 AD, 14 sALS, 92 controls | Evaluate CSF PENK and PDYN peptides as biomarkers of striatal MSN integrity and disease severity in HD compared to other neurodegenerative disorders. | Mean CSF PENK- and PDYN-derived peptide levels were significantly decreased in post-CMD compared to controls and disease comparators. Lower PENK levels correlated with worse motor scores, while lower PDYN correlated with poorer cognitive scores. | Supports CSF PENK and PDYN as candidate markers of striatal MSN dysfunction or loss and clinical severity in HD. | Replicated evidence |
| **Fodale *et al.* (2022) [7]** | tHTT | CSF | 24 pwHD, 6 controls | Develop and validate a novel ultrasensitive SMC immunoassay for polyQ length-independent quantification of tHTT (mHTT + wtHTT) in CSF. | Established a novel SMC assay capable of quantifying tHTT in both control and HD CSF. tHTT levels correlated with mHTT levels but, unlike mHTT, showed no trend with with disease stage in this small cohort. | Enables measurement of total soluble HTT to complement specific mHTT assays. | Exploratory |
| **Ananbeh *et al.* (2022) [8]** | tHTT, mHTT, HTT fragments | Plasma EVs | 1 pwHD, 4 controls | To map the presence of HTT and mHTT forms in plasma-derived small EVs and assess their utility as non-invasive biomarkers. Study used both human and pig plasma. | Full-length mHTT co-isolated with EVs. Trends observed of elevated tHTT in pwHD *vs* controls, but not significant. | Confirms that plasma EVs carry HTT/mHTT and can be isolated via ultracentrifugation. Suggests potential for EV-associated HTT as marker for disease presence, though requiring sensitivity improvements. | Exploratory |
| **Huseby *et al.* (2022) [9]** | RNA transcripts | Whole blood | 27 HD, 488 AD, 397 ALS, 411 FRDA, 298 PD, 80 FTD, 1789 controls | Apply machine learning (random forest) algorithms to public whole blood gene expression datasets to identify transcript biomarkers capable of classifying HD and other neurodegenerative diseases from healthy controls. | Random forest selected 20 blood transcripts, of which the top 10 discriminated HD from controls with high accuracy (AUC 0.89). | Supports whole blood RNA transcripts as candidate biomarkers for HD classification. | Exploratory |
| **Caron *et al.* (2022) [10]** | ALB, APOE, BDNF, C1QB, C1QC, C4B, C7, CHI3L1, CLU, CNR1, CTSD, CYCS, DRD1, DRD2, FAT2, GNAL, IDO1, IGF2, IGHG1, NfL, PDE10A, PDYN, PENK, PPP1R1B, SIGMAR1, TTR CSF | CSF | 16 post_CMD, 8 pre-CMD, 8 controls | Hypothesis-driven analysis of 26 pre-specified CSF proteins using nanLC-PRM-MS to identify novel biomarkers and validate reported ones, as well as to evaluate multi-marker panels for disease stratification. | Replicated changes in NfL, PDYN and PENK and identified novel alterations in C1QB, CNR1, GNAL, IDO1, IGF2, and PPP1R1B across HD status and/or disease stages. PENK was the most discriminant single marker for pwHD *vs* controls (AUC 0.94). Selected multi-marker panels showed high discrimination, including pre-CMD *vs* controls(PENK, ALB, NfL; AUC 1.0) and disease stages. | Supports specific multi-marker CSF panels as candidates for stratifying HD mutation status and disease severity. | Exploratory; replicated evidence (PENK, PDYN); used in interventional studies (NfL) |
| **Anderson *et al.* (2022) [11]** | HVA, miRNA, HTT, p-neurofilaments | CSF, plasma | 6 post_CMD | Investigate safety and tolerability of low-dose nilotinib in post-CMD patients, assessing effects on dopamine metabolites, potential exacerbation of chorea/behavioral symptoms, and CSF miRNA expression. | Found nilotinib was safe and well-tolerated with no worsening of motor or behavioural symptoms. Whole-genome CSF miRNA sequencing found significant alterations in pathways related to autophagy, inflammation and basal ganglia neurotransmission. HVA levels trended towards lowering. | Identifies CSF HVA specific miRNA signatures as potential pharmacodynamic biomarkers for monitoring target engagement (dopamine metabolism) and downstream pathway modulation (autophagy/inflammation) in HD clinical trials. | Exploratory |
| **Parkin *et al.* (2023) [12]** | tHTT, mHTT | Plasma, saliva | 34 pre-CMD, 19 post_CMD, 42 controls | Quantify tHTT and mHTT in matched saliva and plasma samples and investigate associations with clinical measures. | Salivary and plasma HTT levels were uncorrelated. Salivary mHTT was elevated in pre-CMD and post-CMD *vs* controls, and correlated with clinical measures (contrary to plasma mHTT). | Identifies salivary mHTT as a promising non-invasive source for HTT quantification that reflects clinical severity more closely than plasma. | Exploratory (saliva); used in interventional studies (plasma) |
| **Chang *et al.* (2023) [13]** | Lipoprotein subfractions (VLDL, LDL, HDL components) | Plasma | 9 pre-CMD, 24 post-CMD, 20 controls | Investigate alterations in plasma lipoprotein profiles (112 subfractions and components) using NMR spectroscopy to identify metabolic biomarkers of HD. | Significant reductions in selected small dense LDLs and HDL4 components were observed in HD, while VLDL5 free cholesterol was elevated. Selected VLDL5 and HDL4 components correlated with motor and functional scores in post-CMD. LDL5-FC and LDL4-FC discriminated HD from controls (AUC 0.89 each). | Identifies specific lipoprotein components as potential peripheral biomarkers for HD status and clinical severity, reflecting systemic dysregulation of lipid metabolism. | Exploratory |
| **Lin *et al.* (2023) [14]** | Panel of 28 anti-glycan auto-antibodies (including Anti-GD1b, Anti-G17, Anti-G5) | Plasma | 16 pre-CMD, 39 post_CMD, 42 controls | Investigate the potential of anti-glycan auto-antibodies as biomarkers for HD onset and progression using novel ganglioside-focused glycan microarray. | Selected anti-glycan auto-antibodies (specifically IgM) were significantly elevated in pre-CMD compared to controls and post-CMD. Anti-GD1b discriminated pre-CMD from controls (AUC 0.83) and, when combined with age and CAG, discriminated pre-CMD from post-CMD (AUC 0.95). | Supports specific plasma anti-ganglioside auto-antibodies as potential biomarkers of pre-CMD status, potentially suggesting early immune dysregulation. | Exploratory |
| **Li *et al.* (2023) [15]** | NfL | Serum | 90 pre-CMD, 137 post-CMD, 110 controls | Characterise serum NfL trajectories across all disease stages, investigate its relationship with CAG repeat length, and assess its prognostic value using 7.0T MRI and longitudinal clinical data. | Serum NfL was elevated approximately 30 years prior to CMD and correlated with clinical impairment, but did not differ across manifest stages or predict subsequent decline. | Supports serum NfL for early disease monitoring, with potential prognostic value in pre-CMD. | Used in interventional studies |
| **Parkin *et al.* (2023) [16]** | NfL | Plasma | 50 HD-ISS0, 64 HD-ISS1, 63 HD-ISS2, 63 HD-ISS3, 50 controls | Determine the utility of plasma NfL for patient stratification within HD-ISS1. | Plasma NfL levels differed across all stages. Approximately half of Stage 1 participants had NfL levels indicative of predicted CMD within 10 years. | Supports NfL as a tool for enriching HD-ISS1. | Used in interventional studies |
| **Hwang *et al.* (2023) [17]** | NfL, tTau, pTau | Plasma | 6 pre-CMD, 31 post-CMD TFC Stage I-II, 30 post-CMD TFC Stage III-V | Investigate biomarker correlations with clinical severity and functional staging in post-CMD. | NfL was elevated in post-CMD *vs* pre-CMD, but plateaued in post-CMD disease; there was no significant difference between mild and severe groups, and no correlation with TMS or TFC. pTau levels were elevated in post-CMD and continued to rise with disease progression and correlate with TFC worsening. tTau saw no significant group differences or associations. | Supports a complementary biomarker strategy: Plasma NfL for detecting disease onset, plasma pTau for monitoring clinical severity and progression in post-CMD . | Replicated evidence; used in interventional studies (NfL) |
| **Gijs *et al.* (2024) [18]** | mHTT | Tear fluid | 13 pre-CMD, 20 post_CMD, 20 controls | Assess the feasibility of detecting and quantifying mHTT in tear fluid as a novel, non-invasive biomarker source for HD. | mHTT was detectable in all tear samples, with significantly higher levels in pre-CMD and post-CMD compared to controls. Levels correlated with CAG repeat length, disease burden, and clinical motor/functional scores, though no significant difference was found between pre-CMD and post-CMD stages. | Supports tear fluid as a potential non-invasive source for mHTT quantification. | Exploratory |
| **Turner *et al.* (2024) [19]** | NfL | Serum | 28 pre-CMD | Determine whether serum NfL is associated with subjective sleep quality. | No significant association between sleep quality and NfL levels, nor were there significant differences between “good sleep” and “poor sleep” groups. | Subjective sleep quality does not appear to be associated with serum NfL levels. | Used in interventional studies |
| **Cao *et al.* (2024) [20]** | IL-1β, IL-2, IL-4, IL-6, IL-8, IL-10, IL-12p70, IL-13, TNF-α, IFN-γ | Plasma | 21 pre-CMD, 83 post-CMD, 31 controls | Use data-driven clustering (PCA and k-means) to identify clinical subtypes of HD and characterise them using plasma cytokine levels and structural MRI. | Identified distinct clinical clusters. Cluster 1 (severe motor/non-motor symptoms, lowest brain volume) had significantly higher plasma IL-2 levels. IL-2 correlated negatively with functional capacity and cognitive scores. IFN-γ correlated with CAG repeat length. | Identifies plasma IL-2 as a potential biomarker for severe, hypokinetic-rigid HD subtype characterised by extensive brain atrophy and cognitive decline, linking neuroinflammation to specific clinical phenotypes. | Exploratory |
| **Martinez-Horta *et al.* (2024) [21]** | NfL, tTau, pTau-231 | CSF | 22 pre-CMD, 30 post-CMD | Investigate the association between tau pathology and NfL with specific cognitive phenotypes and brain atrophy patterns to understand mechanisms underlying clinical heterogeneity in HD. | All biomarkers were elevated in post-CMD compared with pre-CMD. High tTau and pTau-231 levels showed additional associations with specific deficits and cortical thinning, including posterior regions, not observed for NfL. | Suggests CSF tTau and pTau-231 may complement NfL in characterising cognitive heterogeneity in HD. | Replicated evidence; used in interventional studies (NfL) |
| **Korpela *et al.* (2024) [22]** | GFAP, Aβ42 | CSF | 13 pre-CMD, 28 post-CMD, 20 controls | Investigate the utility of Aβ42 (protein aggregation) and GFAP (astrocyte activation) as potential biomarkers for HD onset and progression. | Aβ42 levels did not differ significantly between groups or correlate with clinical measures. GFAP was significantly elevated in post-CMD, correlated strongly with lower cUHDRS scores across pwHD, and higher 5-year onset risk in pre-CMD. | Identifies CSF GFAP as a potential biomarker of disease severity and astrocyte activation that predates CMD, while indicating limited biomarker potential for Aβ42 in this cohort. | Replicated evidence |
| **Parkin *et al.* (2024) [23]** | NfL | Plasma | 37 HD-ISS0-1, 41 HD-ISS2-3, 30 controls | Investigate the 5-year longitudinal trajectory of plasma NfL across the HD spectrum to determine its relationship with clinical progression. | NfL levels increased linearly in early disease (HD-ISS0-1) correlating with cognitive impairment (SDMT), then showed a quadratic acceleration in later stages (HD-ISS3) where it correlated with both motor and cognitive deficits. | Identifies plasma NfL as a dynamic biomarker that increases linearly in early-stage disease, followed by an acceleration, supporting its use to enrich early-stage (HD-ISS1) cohorts for clinical trials. | Used in interventional studies |
| **Machacek *et al.* (2024) [24]** | NfL | CSF | 20 pre-CMD, 40 post-CMD, 20 controls | Develop a semi-mechanistic model to quantify the relationship between CSF NfL dynamics, CSF flow rates and the rate of whole brain atrophy. | The model demonstrated that CSF NfL level reflect the rate of neurodegeneration rather than the extent (total volume lost). NfL rise is driven by accelerating atrophy and reducedclearance in HD. | Establishes CSF NfL as a specific quantitative marker for the rate of neurodegeneration, validating its use to monitor disease acceleration and providing a mechanistic link between tissue damage and fluid levels. | Used in interventional studies |
| **Chiang *et al.* (2024) [25]** | Panel of 16 bile acids (including GCDCA, GUDCA, iso-LCA) | Plasma | 9 pre-CMD, 24 post-CMD, 20 controls | Investigate variations in plasma bile acid profiles to identify metabolic biomarkers for diagnosing and staging HD. | Plasma GCDCA and GUDCA were significantly elevated in pwHD, while iso-LCA was reduced. Summed concentrations of bile acids classified as neuroprotective were elevated in pre-CMD, whereas those classified as neurotoxic were elevated in post-CMD, each compared with controls. | Supports specific plasma bile acids as potential biomarkers of HD status and disease stage. | Exploratory |
| **Voysey *et al.* (2024) [26]** | NfL | Serum | 21 pre-CMD, 14 controls | Investigate 14-year longitudinal dynamics of serum NfL to determine its prognostic value in predicting disease onset and clinical progression. | Baseline NfL levelsdiscriminated converters from non-converters, with converters showing higher levels and faster rates of increase. Higher rates of change of NfL also predicted greater severity in motor deficits and cognitive decline. | Identifies serum NfL as a predictive biomarker capable of discriminating individuals at risk of symptomatic onset over a decade in advance. | Used in interventional studies |
| **Herrero-Lorenzo *et al.* (2024) [27]** | sRNAs (miRNA, tRFs) | Plasma EVs | 21 pre-CMD, 20 post-CMD, 22 controls | Characterise sRNA profiles within plasma EVs to identify potential biomarkers of pre-CMD changes. | Specific sRNAswere significantly downregulated in plasma EVs of pwHD, including pre-CMD. For some, levels correlated with pre-CMD cognitive decline. | Identifies EV-associated sRNAs as potential biomarkers of early HD changes that may complement NfL, with preliminary evidence for monitoring pre-CMD progression. | Exploratory |
| **Anderson *et al.* (2025) [28]** | NfL | Plasma | 9 pwHD, 12 HDL2, 9 controls | Evaluate plasma NfL as a biomarker for HDL2 by comparing levels in pwHD, HDL2 and controls. | Levels elevated in pwHD compared to controls, with excellent diagnostic discrimination (AUC 0.99). Levels were also elevated in HDL2 with excellent discriminatory power. | Supports NfL as a biomarker for HD (and HDL2). | Used in interventional studies |
| **Voysey *et al.* (2025) [29]** | NfL | Serum | 28 pre-CMD, 21 controls | Determine if serum NfL elevation correlates with objective sleep fragmentation (WASO) and cognitive decline over a 12-year longitudinal period. | Serum NfL levels were significantly elevated in converters (pre-CMD to prodromal or post-CMD). Higher serum NfL concentrations strongly correlated with increased WASO. | Supports serum NfL as a marker of disease activity associated with sleep maintenance disturbance in HD. | Used in interventional studies |
| **Bamford *et al.* (2025) [30]** | NfL, tTau, GFAP, YKL-40 | CSF, plasma, saliva | 21 pwHD; 26 pre-CMD, 49 post-CMD, 20 controls | Determine how well biomarker levels in CSF correlate with peripheral levels (plasma and saliva) in HD, and evaluate saliva as a non-invasive source for tracking disease symptoms. | NfL and GFAP showed a positive correlation between CSF and plasma, but a negative correlation between CSF and saliva. In the larger validation cohort, plasma NfL negatively correlated with salivary NfL in pwHD, but positively correlated in healthy controls. Salivary NfL was negatively correlated with chorea in post-CMD. Salivary tTau correalted positively with cognitive measures in pre-CMD. | Supports salivary NfL and tTau as potential non-invasive biomarkers for specific stages of HD. | Exploratory |
| **McGarry & Moaddel (2025) [31]** | CSF proteome | CSF | 8 post-CMD | Cross-sectional proteomic analysis to identify biomarkers correlating with TFC and disease severity. | Identified 47 proteins correlating with functional capacity, including inflammatory markers, neurodegeneration markers and NAD+ metabolism regulators. | Identifies a broad panel of CSF proteins with potential for enrichment utility. | Exploratory |
| **Scahill *et al.* (2025) [32]** | NfL, PENK, YKL-40, SER | CSF, Blood | 57 HD-ISS0-1, 46 controls | Investigate the 4.5-year longitudinal changes in fluid and imaging biomarkers and blood somatic expansion in young adult pwHD (~23 predicted YTO) to identify early drivers of pathology. | Despite no clinical decline at HD-ISS0-1 compared to controls, pwHD showed elevated baseline CSF NfL and YKL-40 with faster longitudinal increases, decreases in CSF PENK, and caudate and putamen atrophy. Blood SER increased longitudinally and was associated with striatal atrophy after adjustment for age and inherited CAG length. | Supports a role for somatic CAG expansion in early neurodegeneration and the potential use of CSF NfL, PENK and blood SER as biomarkers for future clinical trials in early-stage HD. | Replicated evidence; used in interventional studies (NfL) |
| **Heim *et al.* (2025) [33]** | NfL, GFAP | Serum | 13 pre-CMD, 31 post-CMD, 19 controls | Evaluate the utility of serum NfL and GFAP in tracking different stages of HD and correlate them with clinical progression markers. | Serum NfL was significantly elevated in all HD stages, including pre-CMD. NfL showed superior discriminatory accuracy in identifying pwHD from controls compared to GFAP (AUC 0.99 *vs* 0.61). GFAP was only elevated in advanced HD, where it was superior to NfL for distinguishing advanced stages (AUC 0.97 *vs* 0.79). | Establishes serum NfL as the preferred marker for early detection (neuroaxonal damage), while serum GFAP (astrogliosis) has potential to serve as a marker for late-stage progression. | Replicated evidence; used in interventional studies (NfL) |
| **Alpaugh *et al.* (2025) [34]** | t-tau, NTA-tau, p-tau (181, 231, 199) | Plasma, platelets, PBMCs | Plasma: 16 pre-CMD, 48 post-CMD, 61 controls; platelets: 10 pre-CMD, 23 post-CMD, 29 controls; PBMCs: 9 pre-CMD, 43 post-CMD, 45 controls. | Evaluate tau species in distinct blood compartments (plasma, platelets, PBMCs) to determine their utility as peripheral biomarkers for HD severity and cognition. | Plasma NTA-tau and pTau181 correlated with disease stage and poorer cognitive performance. Platelet tTau in platelets was significantly elevated in post-CMD *vs* controls and correlated with disease stage and TFC. | Supports platelet tTau as a potential peripheral biomarker of HD severity and functional impairment. | Exploratory (platelets, PBMC); replicated evidence (plasma) |
| **Leckey et al. (2024) [35]** | NfL | CSF | 10 post-CMD, 65 other diseases, 10 controls | Characterise NfL species in CSF and validate a cost-effective targeted IP-MS assay. | HD patients showed highest NfL concentrations of all disease groups. | Validates a cost-effective, sensitive IP-MS alternative to immunoassay for monitoring NfL levels. | Used in interventional studies |
| **Owen *et al.* (2025) [36]** | GFAP, tTau, NfL | Serum | 21 pre-CMD, 14 controls | Investigate the 14-year longitudinal dynamics and prognostic value of serum GFAP and tTau in pre- and transitional-HD, comparing them with NfL. | Neither GFAP nor tTau showed significant longitudinal changes or group differences between converters and non-converters. While baseline GFAP predicted later motor severity, neither biomarker could discriminate phenoconversion risk, unlike NfL in the same cohort. | Suggests that serum GFAP and tTau are inferior to NfL as prognostic biomarkers for pre-CMD/early post-CMD. | Replicated evidence; used in interventional studies (Nfl) |
| **Qian *et al.* (2025) [37]** | Gut microbiota (Bacteroides, Faecalibacterium), Serum metabolites (dimethisterone, vanillin) | Faeces, Serum | 26 pwHD (11 faecal, 26 serum), 23 controls (16 faecal, 23 serum) | Perform a multi-omics analysis to identify key gut microbiota and serum metabolites associated with HD pathogenesis via the gut-brain axis. | HD patients showed altered gut microbiota composition and 244 differential serum metabolites linked to lipid/nucleotide metabolism. Integrated analysis of microbiota and metabolites discriminated HD from controls (AUC = 1.0). | Identifies specific gut microbiota and serum metabolites as potential biomarkers for HD. | Exploratory |
| **Khetarpal *et al.* (2025) [38]** | Kynurenine pathway metabolites (3-OH-KYN, QA, KYN, KYNA, TRP, AA) | CSF, Plasma | 141 post-CMD (126 early, 15 moderate), 75 controls | Investigate the hypothesis of kynurenine dysregulation in HD by quantifying 6 key metabolites in CSF and plasma using LC-MS/MS methods. | No significant differences were found in the concentrations of any metabolite between pwHD and controls in either CSF or plasma. Metabolite levels showed no convincing associations withclinical severity after multiple-testing correction, though several CSF metabolites were positively associated with age. | Provides no evidence of kynurenine pathway dysregulation in HD in CSF or plasma, suggesting these metabolites may not be useful biomarkers in this context. | Exploratory |
| **Estevez-Fraga *et al.* (2025) [39]** | NfL | Plasma | 252 pwHD, 260 controls | To map brain connectivity changes across HD stages and link them to biological mechanisms and neuroaxonal damage. | Plasma NfL levels correlated significantly with brain hypoconnectivity in late pre-CMD and post-CMD, but not with hyperconnectivity in early pre-CMD. | Plasma NfL levels provide mechanistic insight, confirming that connectivity loss observed in MRI reflects true underlying neurodegeneration. | Used in interventional studies |
| **Coleman & Touze *et al.* (2025) [40]** | NfL, GFAP, tTau | Plasma, serum | 57 pwHD, 34 MS, 7 ALS, 11 PD, 54 controls; 64 pwHD, 57 controls | To technically validate a remote collection method for quantifying neuropathology biomarkers, assessing equivalence of capillary and venous blood in plasma and serum, and biomarker stability. | Capillary NfL and GFAP concentrations were highly correlated with and equivalent to venous levels across both plasma and serum. NfL demonstrated high stability over a 7-day processing delay, while GFAP levels declined and tTau levels were highly variable. NfL was elevated in pwHD compared to controls, with CAG-dependence. | Validates finger-prick collection as a reliable, minimally invasive alternative to venepuncture for NfL quantification. | Replicated evidence; used in interventional studies (NfL) |
| **Farag *et al.* (2025) [41]** | PENK, NfL | CSF, plasma | 54 HD-ISS0-1, 34 controls | Investigate whether baseline CSF PENK level predicts regional brain atrophy over time compared to NfL, and to evaluate the ability of these biomarkers to discriminate between early disease stages. | Lower baseline CSF PENK significantly predicted grey and white matter atrophy, predominantly in the striatum, while higher baseline CSF NfL predicted widespread white matter loss. CSF PENK (AUC 0.71) outperformed CSF NfL (AUC 0.66) in discriminating HD-ISS 0 *vs* 1. | Supports CSF PENK as a mechanistically specific biomarker for striatal MSN integrity that changes decades prior to motor diagnosis, with potential for enriching clinical trial cohorts in the earliest stages of HD. | Replicated evidence; used in interventional studies (NfL) |
| **Meda *et al.* (2025) [42]** | AINX, NfL | CSF | 9 pwHD, 15 AD, 4 CBS, 5 SD, 18 DLB, 9 MS, 2 bvFTD, 5 controls; 20 AD, 20 controls | To develop and validate a highly sensitive Simoa immunoassay for AINX—a CNS specific intermediate filament—and compare its diagnostic performance and correlation with NfL across various neurodegenerative diseases. | AINX levels were significantly elevated in pwHD compared to controls. AINX showed a strong positive correlation with NfL, though absolute concentrations were lower than NfL. | Supports AINX as a measurable, stable, and CNS-specific biomarker for neurodegeneration, mirroring NfL trends but with potential for distinct clinical utility in distinguishing brain-specific damage from peripheral nerve injury. | Exploratory; used in interventional studies (NfL) |

**Abbreviations:** TDP-43, TAR DNA-binding protein 43; pwHD, persons with Huntington’s disease; HD, Huntington’s disease; mHTT, mutant huntingtin; NfL, neurofilament light; pre-CMD, before clinical motor diagnosis of HD; post-CMD, after clinical motor diagnosis of HD; PIN, prognostic index normalised; YTO, years to onset; CAG, cytosine-adenine-guanine; JOHD, juvenile-onset Huntington’s disease; AUC, area under the curve; CSF, cerebrospinal fluid; PENK, proenkephalin; PDYN, prodynorphin; PD, Parkinson’s disease; AD, Alzheimer’s disease; sALS, sporadic amyotrophic lateral sclerosis; MSN, medium spiny neuron; tHTT, total huntingtin; SMC, single molecule counting; polyQ, polyglutamine; wtHTT, wild-type huntingtin; FRDA, Friedreich’s ataxia; FTD, frontotemporal dementia; RNA, ribonucleic acid; ALB, albumin; APOE, apolipoprotein E; BDNF, brain-derived neurotrophic factor; C1QB, complement C1q subcomponent subunit B; C1QC, complement C1q subcomponent subunit C; C4B, complement C4B; C7, complement component 7; CHI3L1, chitinase-3-like protein 1; CLU, clusterin; CNR1, cannabinoid receptor 1; CTSD, cathepsin D; CYCS, cytochrome c; DRD1, dopamine receptor D1; DRD2, dopamine receptor D2; FAT2, FAT atypical cadherin 2; GNAL, guanine nucleotide-binding protein G(olf) subunit alpha; IDO1, indoleamine 2,3-dioxygenase 1; IGF2, insulin-like growth factor 2; IGHG1, immunoglobulin heavy constant gamma 1; PDE10A, phosphodiesterase 10A; PPP1R1B, protein phosphatase 1 regulatory inhibitor subunit 1B; SIGMAR1, sigma non-opioid intracellular receptor 1; TTR, transthyretin; nanoLC-PRM-MS, nanoflow liquid chromatography-parallel reaction monitoring-mass spectrometry; HVA, homovanillic acid; miRNA, microRNA; VLDL, very low-density lipoprotein; LDL, low-density lipoprotein; HDL, high-density lipoprotein; NMR, nuclear magnetic resonance; IgM, immunoglobulin M; HD-ISS, Huntington’s disease integrated staging system; CMD, clinical motor diagnosis; tTau, total tau; pTau, phosphorylated tau; TFC, total functional capacity; TMS, total motor score; IL, interleukin; TNF-α, tumor necrosis factor alpha; IFN-γ, interferon gamma; PCA, principal component analysis; pTau-231, phosphorylated tau at threonine 231; GFAP, glial fibrillary acidic protein; Aβ42, amyloid beta 42; cUHDRS, composite unified Huntington’s disease rating scale; SDMT, symbol digit modalities test; GCDCA, glycochenodeoxycholic acid; GUDCA, glycoursodeoxycholic acid; iso-LCA, iso-lithocholic acid; sRNA, small RNA; tRFs, tRNA-derived fragments; EVs, extracellular vesicles; SWRT, Stroop word reading test; HDL2, Huntington disease-like 2; WASO, wake after sleep onset; YKL-40, chitinase-3-like protein 1; VIM, vimentin; NAD+, nicotinamide adenine dinucleotide; SIRT2, sirtuin 2; SIRT5, sirtuin 5; ECM, extracellular matrix; SER, somatic expansion rate; NTA-tau, N-terminal assay tau; PBMC, peripheral blood mononuclear cell; MMSE, mini-mental state examination; 3-OH-KYN, 3-hydroxykynurenine; QA, quinolinic acid; KYN, kynurenine; KYNA, kynurenic acid; TRP, tryptophan; AA, anthranilic acid; LC-MS/MS, liquid chromatography-tandem mass spectrometry; MS, multiple sclerosis; AINX, alpha-internexin; CBS, corticobasal syndrome; SD, semantic dementia; DLB, dementia with Lewy bodies; bvFTD, behavioural variant frontotemporal dementia; IP-MS, immunoprecipitation mass spectrometry.

**Supplementary Table 3**. Main study findings of imaging biomarkers in Huntington’s disease from search between 1st January 2022 and 6th October 2025. Biomarker status indicates the current level of evidence and translational development for each candidate within the specified pipeline and analytical technique. Exploratory biomarkers are supported by early or limited evidence. Replicated biomarkers have evidence across more than one independent cohort, study or analytical context. Biomarkers used in interventional studies have been incorporated into clinical trials as either exploratory or secondary endpoints. Currently no biomarkers are approaching regulatory qualification for a defined context of use.

| **First author (Year)** | **Modality** | **N** | **Aim** | **Key findings** | **Biomarker relevance** | **Biomarker status** |
| --- | --- | --- | --- | --- | --- | --- |
| **Zeun *et al.* (2022) [43]** | Diffusion MRI | 126 pre-CMD, 138 controls | Determine when selective basal ganglia WM loss begins in relation to motor onset, and which striatal/thalamic tracts are most vulnerable | WM tracts of cortico–basal ganglia sub-regions are largely preserved ~25 years before onset, with progressive, regionally selective WM loss emerging closer to predicted clinical onset (motor and limbic/fronto-striatal tracts most vulnerable) | **Suggests** a long preclinical window where WM remains relatively intact; supports targeted use of WM measures as progression markers nearer onset. Highlights importance of tract-specific rather than global WM metrics | Replicated evidence |
| **Barrios-Martinez *et al*. (2022) [44]** | Differential tractography | 4 pre-CMD, 12 post-CMD | Assess differential tractography as a dynamic imaging biomarker of neurodegeneration in HD | Differential tractography identified WM segments with reduced anisotropy consistent with degeneration; changes could be detected in pre-CMD and post-CMD and mapped at the individual level | Demonstrates feasibility of differential tractography as a dynamic, individual-level WM biomarker in HD; limited by small sample and methodological pilot nature | Exploratory |
| **Lavrador *et al*. (2022) [45]** | Structural and diffusion-weighted MRI | 14 pre-CMD, 11 post-CMD, 18 controls | Classify HD stages using SVMs and combined structural + diffusion features, and compare feature-selection strategies | Multimodal imaging achieved good classification of controls *vs* pre-CMD *vs* early post-CMD combining modalities outperformed single-modality models. Fronto-striatal and WM features were most informative in classifying | Supports multimodal MRI feature sets as useful for disease-stage classification/stratification. Sample size small; needs validation for clinical use | Exploratory |
| **Schmid *et al*. (2022) [46]** | OCT | 24 pre-CMD, 38 controls | Assess whether OCT detects retinal neurodegeneration in far-from-CMD | No significant differences in retinal layer thickness between pre-CMD and controls | Indicates no detectable OCT changes very far from onset; suggests OCT is not a sensitive early biomarker in far-from-CMD | Exploratory |
| **Tan *et al*. (2022) [47]** | Structural MRI | 26 pre-CMD, 26 post-CMD, 24 controls | Map longitudinal cortical surface changes and folding alterations in HD | Significant longitudinal decline in local gyrification (especially right lateral occipital and precentral regions) during post-CMD; subtler or absent changes in early post-CMD/pre-CMD | Showed cortical folding/gyrification measures change over time, particularly later in the disease. Potential biomarker of cortical involvement, but less sensitive than striatal volumetry in early disease | Exploratory |
| **Casella *et al*. (2022) [48]** | Ultra-strong gradient diffusion MRI and magnetisation transfer ratio (MTR) | 25 pre-CMD, 25 controls | Disentangle contributions of axon density *vs* myelin/MT to WM changes in pre-CMD | WM differences in pre-CMD were driven primarily by mutation-related MTR changes, not restricted diffusion measures | Suggests myelin/MT alterations rather than axon loss underlie early WM differences; points to MTR as a sensitive pre-CMD biomarker. | Exploratory |
| **Tan *et al.* (2022) [49]** | Diffusion tensor MRI | 25 pre-CMD, 27 post-CMD 25 controls | Investigate longitudinal changes in frontal cortico-striatal WM tracts in HD | Progressive microstructural changes (reduced FA/increased RD) in tracts connecting striatum with prefrontal and motor regions; alterations more pronounced with advancing disease and associated with clinical measures | Supports fronto-striatal WM DTI metrics as longitudinal biomarkers of progression, complementary to volumetry | Replicated evidence |
| **Ponomareva *et al*. (2023) [50]** | EEG and rs-fMRI connectivity | 20 pre-CMD, 24 post-CMD, 24 controls | Identify neurophysiological hallmarks of HD progression by examining EEG and rs-fMRI connectivity and their inter-relationships | Pre-CMD showed reduced EEG power at the theta–alpha border/slow alpha and altered rs-fMRI connectivity in frontal and putamen-cortical networks; early post-CMD showed more pronounced changes. Relationships between alpha power and rs-FC differed from controls | Demonstrates combined EEG–fMRI measures capture early functional network disruption and progression, offering multimodal functional biomarkers; needs longitudinal validation | Exploratory |
| **Yao *et al*. (2023) [51]** | 7T QSM | 5 pwHD, 5 controls | Compare multiple QSM dipole inversion algorithms for iron-sensitive imaging at 7T and assess their performance in HD | Single-step QSM methods produced susceptibility values most correlated with reference iron measures and better distinguished pre-CMD from controls than other algorithms | Emphasises that pipeline choice critically affects QSM-derived iron biomarkers; provides methodological basis for using single-step QSM in HD biomarker work | Exploratory |
| **Dusek *et al*. (2023) [52]** | OCT | 41 pwHD, 41 controls | Evaluate whether retinal structure and function are affected in HD and whether OCT is a useful biomarker | Small, diffuse reductions in global RNFL and macular measures in pwHD, but effect sizes were small and not suitable for monitoring progression; no strong functional retinal deficits | Suggests retinal OCT shows subtle abnormalities in post-CMD but lacks sensitivity/specificity as a progression biomarker | Exploratory |
| **Hu *et al*. (2023) [53]** | Diffusion tensor MRI | 463 pre-CMD, 72 post-CMD, 194 controls | Characterise regional and temporal vulnerability of deep GM and WM using DTI across pre- and post-CMD | Identified distinct regional and temporal patterns of DTI change in deep GM (striatum, thalamus) and WM; microstructural alterations increased with CAP/disease burden and across disease stages | Provides strong evidence that DTI metrics in deep GM and WM are sensitive to HD progression and may complement volume measures for experimental therapeutics | Replicated evidence |
| **Lemoine *et al*. (2023) [54]** | Structural MRI | 25 pre-CMD, 60 post-CMD, 53 controls | Investigate the role of the striatum in time production and its relationship to disease progression | Abnormal time production performance corresponded with striatal degeneration over time; timing deficits worsened with disease progression | Links striatal structural change with a specific cognitive/behavioural phenotype (time production), highlighting a potential combined imaging–behaviour biomarker; primarily neuropsychological focus | Exploratory |
| **Delva *et al.* (2023) [55]** | PET (18F-FDG, SV2A), sMRI | 16 precmd, 16 early post-CMD, 16 controls | Compare longitudinal sensitivity of MRI and PET measures for tracking disease progression in early HD | Striatal volumes showed the grated longitudinal sensitivity to change; PET detected synaptic and metabolic deficits but with small effect sizes over 2 years | Strengthens sMRI as a sensitive prognostic biomarker. | Replicated evidence |
| **Stoebner *et al*. (2023) [56]** | Structural MRI | 284 pwHD, 111 controls | Perform a comprehensive cortical shape analysis in HD using multiple morphometric metrics | Confirmed widespread cortical thinning and additional changes in sulcal depth and gyrification; some regional shape features were associated with cognitive scores and disease burden | Suggests multi-metric cortical shape analysis may capture subtle cortical involvement and relate to cognition, but striatal volumetry remains more established | Exploratory |
| **Estevez-Fraga *et al*. (2023) [57]** | Diffusion tensor MRI | 67 pre-CMD, 10 post-CMD, 67 controls | Examine progressive alterations in WM microstructure across the HD time course | Demonstrated significant longitudinal WM microstructural changes; affected areas and number of altered metrics increased with advancing disease stage | Strong evidence that WM microstructure shows progressive, stage-dependent change, supporting diffusion MRI as a longitudinal biomarker across pre- and early post-CMD | Replicated evidence |
| **Wilkes *et al.* (2023) [58]** | Structural MRI | 25 pre-CMD, 27 post-CMD, 25 controls | Investigate hippocampal morphology in HD and implications for plasticity/pathogenesis | No clear group differences in overall hippocampal volume; localised shape differences and potential region-specific expansions/contractions were seen, with limited clinical associations | Indicates hippocampal volume is not a strong biomarker, but subtle shape changes may reflect plasticity or region-specific vulnerability | Exploratory |
| **Wilkes *et al.* (2023) [59]** | Structural MRI | 25 pre-CMD, 27 post-CMD, 25 controls | Map spatiotemporal progression of striatal morphology and relate it to clinical measures | Found localised striatal shape changes over time in pre-CMD and post-CMD *vs* controls; particular regions of the striatum showed progressive thinning/deflation linked to motor and cognitive measures | Provides detailed spatiotemporal striatal shape signatures that track progression and clinical status; supports use of surface-based striatal metrics as biomarkers | Exploratory |
| **Demir & Rosas (2024) [60]** | Diffusion MRI | 25 pwHD, 25 controls | Evaluate changes in interhemispheric connectivity using dTDPs | Demonstrated altered interhemispheric connectivity patterns in pwHD compared with controls, suggesting disruption of callosal and other interhemispheric tracts | Shows interhemispheric dTDP metrics are sensitive to disease-related WM changes; promising connectivity biomarker but requires replication and longitudinal data | Exploratory |
| **Ghofrani-Jahromi *et al.* (2024) [61]** | sMRI with machine learning | 451 pwHD | Develop machine-learning models for prognostic enrichment in early HD | Models predicted 3-year ventricular enlargement more accurately than clinical measures alone and stratified pwHD into fast *vs* slow progressors | Supports use of sMRI measures as prognostic biomarkers for stratification and enrichment in clinical trials | Replicated evidence |
| **Yin *et al.* (2024) [62]** | Diffusion kurtosis MRI | 22 pwHD, 14 controls | Assess whether DKI provides greater sensitivity than DTI for detecting early white matter microstructural changes in HD | Widespread reductions in mean kurtosis detected in more regions than FA; diffusion metrics strongly correlated with disease burden | Evidence that diffusion MRI, particularly DKI as a candidate prognostic biomarker of early white matter pathology | Replicated evidence |
| **van Wamelen *et al.* (2024) [63]** | Multi-modal PET, multi-modal MRI | Target of ~60-80 pwHD and controls | Characterise longitudinal changes in molecular PET targets and MRI measures in early HD | Study protocol describing baseline, 1-year and 2-year multi-modal imaging to assess striatal and cortical molecular alterations | Aiming to evaluate molecular PET and MRI as candidate pharmacodynamic and prognostic biomarkers in early post-CMD | Exploratory |
| **Barrett *et al.* (2024) [64]** | sMRI with predictive modelling | 630 pwHD | Optimise screening and eligibility for intrastriatal interventions using predictive models | Logistic models combining striatal volume, age and CAG repeat length predicted surgical eligibility with high accuracy | Demonstrates sMRI’s potential use to support trial feasibility and participant selection | Replicated evidence |
| **Cao *et al*. (2024) [20]** | Structural MRI | 21 pre-CMD, 83 post-CMD, 31 controls | Identify and validate HD subtypes based on clinical features, neuroimaging and cytokine profiles | Unsupervised analyses identified distinct HD subtypes with differing clinical manifestations, neuroimaging patterns (e.g. differing degrees/regions of atrophy) and cytokine signatures | Suggests imaging combined with inflammatory markers can define biologically distinct HD subtypes, relevant for stratification | Exploratory |
| **Yao *et al*. (2024) [65]** | 7T QSM and Diffusion MRI | 28 pre-CMD, 15 post-CMD, 14 controls | Assess iron accumulation and microstructural alterations in subcortical nuclei (striatum, thalamus, globus pallidus) in HD using ultra-high-field 7T MRI | Post-CMD showed increased susceptibility (iron) and microstructural abnormalities in caudate, putamen and globus pallidus; pre-CMD groups showed intermediate changes, with near‑onset carriers more affected than far‑from‑onset | 7T iron‑sensitive and microstructural measures in subcortical nuclei are sensitive to stage and may serve as early biomarkers, particularly in pre-CMD individuals close to onset | Exploratory |
| **De Paepe *et al.* (2025) [66]** | rs-fMRI | 63 pwHD, 35 controls | Determine whether distinct striato-cortical connectivity patterns predict different clinical profiles | PCA defined clinical profiles (motor–cognitive *vs* behavioural) associated with distinct patterns of striato‑cortical functional connectivity and structural alterations. Some pwHD showed more motor‑dominant, others more cognitive/behavioural network disruption | Demonstrates striato-cortical connectivity patterns can predict clinical heterogeneity, supporting use of rs-fMRI connectivity as a stratification biomarker | Replicated evidence |
| **Scahill *et al.* (2025) [32]** | sMRI | 57 HD-ISS (0-1), 46 controls | Characterise longitudinal brain volume changes in far-from-onset HD and assess whether peripheral SER predicts early neurodegeneration | Progressive caudate and putamen atrophy detectable over 4.5 years despite volumes remaining within normal range at baseline. Annualised atrophy rates diverged significantly from controls | Confirms striatal volumetric MRI as a sensitive early prognostic biomarker in far-from-onset HD; imaging changes were predicted by longitudinal increases in SER, supporting integration of MRI with mechanistic biomarkers for early-stage trials | Trial-endpoint |
| **Giannoula *et al.* (2025) [67]** | sMRI with unsupervised machine learning (DTW clustering) | 22 pre-CMD, 25 post-CMD | Identify distinct disease trajectories using longitudinal imaging and clinical data | Identified three progression clusters (slow, intermediate and fast) distinguished by striatal atrophy rates and clinical decline patterns | Highlights longitudinal sMRI’s use in capturing heterogeneity in disease progression as a prognostic biomarker | Replicated evidence |
| **Abeyasinghe *et al.* (2025) [68]** | Structural MRI | 657 pwHD, 284 controls | Test whether brain age gap can stratify disease stage and severity in HD | pwHD showed increased brain-age gap *vs* controls; larger gaps associated with more advanced disease and worse clinical measures | Provides evidence that MRI-based brain age is a useful stratification and progression biomarker in HD, potentially valuable for trials | Exploratory |

**Abbreviations:** MRI, magnetic resonance imaging; pre-CMD, before clinical motor diagnosis of HD; post-CMD, after clinical motor diagnosis of HD; WM, white matter; SVM, support vector machine; OCT, optical coherence tomography; MTR, magnetisation transfer ratio; FA, fractional anisotropy; RD, radial diffusivity; EEG, electroencephalography; rs-fMRI, resting-state functional magnetic resonance imaging; rs-FC, resting-state functional connectivity; pwHD, persons with Huntington’s disease; RNFL, retinal nerve fibre layer; DTI, diffusion tensor imaging; GM, grey matter; CAP, CAG-age product score; QSM, quantitative susceptibility mapping; 7T, 7-Tesla; PCA, principal component analysis; dTDPs, diffusion tensor distribution profiles; DKI, diffusion kurtosis imaging; PET, positron emission tomography; FDG, fluorodeoxyglucose; SV2A, synaptic vesicle glycoprotein 2A; CAG, cytosine-adenine-guanine; DTW, dynamic time warping.

Historical evidence of fluid and imaging biomarkers in Huntington’s disease

**Supplementary Table 4**. Key historical evidence of the fluid biomarkers in Huntington’s disease discussed in this review prior to 1st January 2022: NfL, mHTT, PENK, PDYN and SER. Evidence is limited to human studies.

| **TRACK-HD** | | | | |
| --- | --- | --- | --- | --- |
| **First author** | **Year** | **N** | **Key findings** | **Historical significance** |
| Byrne *et al.* [69] | 2017 | 104 pre-CMD, 97 post-CMD, 97 controls; validation in HD-CSF cohort: 23 pwHD, 14 controls | Plasma NfL rose with disease stage and CAG repeat length, and was highly correlated with CSF NfL and brain atrophy by MRI. Baseline plasma NfL predicted clinical onset within 3 years in pre-CMD and tracked cognitive decline. | Established NfL as the first robust blood biomarker for HD. |
| Johnson *et al.* [70] | 2018 | 120 pre-CMD, 123 post-CMD | Baseline plasma NfL predicted caudate, putamen, white matter and grey matter atrophy. | Confirmed plasma NfL reflects neurodegeneration within the specific brain regions that degenerate in HD. |
| **HD-CSF** | | | | |
| **First author** | **Year** | **N** | **Key findings** | **Historical significance** |
| Wild *et al.* [71] | 2015 | 9 pwHD, 3 controls; 30 pwHD, 10 controls | Demonstrated the mHTT could be quantified in the CSF of pwHD, and that levels correlate with disease stage and clinical severity. | Established CSF mHTT as a biomarker in HD. |
| Byrne *et al.* [72] | 2018 | 20 pre-CMD, 40 post-CMD, 20 controls | Demonstrated that while CSF mHTT detects specific pathology, NfL (ins CSF and plasma) correlates more strongly with clinical severity and MRI markers of severity. | Differentiated the utility of the two markers: mHTT as a specific pharmacodynamic marker of target engagement, and NfL as a general prognostic marker. |
| Rodrigues & Byrne *et al.* [73] | 2020 | 20 pre-CMD, 40 post-CMD, 20 controls | mHTT rises linearly, while NfL follows a sigmoidal curve. Both blood and CSF NfL offered stronger prognostic capability than mHTT for brain atrophy and clinical decline. | Validated mHTT as the ideal marker for target engagement (due to linear stability) and NfL as the superior biomarker for prognosis. |
| **HD Young Adult Study (HD-YAS)** | | | | |
| **First author** | **Year** | **N** | **Key findings** | **Historical significance** |
| Scahill *et al.* [74] | 2020 | 64 pre-CMD, 67 controls | Despite normal cognitive, motor and psychiatric function, and largely normal brain structure (small reduction in putamen only), CSF NfL was significantly elevated, detecting neuronal damage approximately 24 years before predicted onset. CSF NfL outperformed plasma NfL at this very early stage. | Concluded that CSF NfL is the most sensitive marker of early disease progression, detectable decades prior to clinical symptom onset. |
| **Other Cohorts** | | | | |
| **First author** | **Year** | **N** | **Key findings** | **Historical significance** |
| Tabrizi *et al.* [75] | 2019 | 46 pwHD | Randomised, double blinded, multiple-ascending-dose, Phase I/IIa trial of ASO tominersen, which resulted in dose-dependent lowering of mHTT protein in CSF. | First proof-of-concept in humans that a drug could lower the specific disease-causing mHTT protein, as measured in CSF. |
| GeM-HD Consortium [76] | 2019 | 9064 pwHD (ENROLL-HD & EHDN Registry) | Established that uninterrupted CAG repeat length, rather than polyQ peptide length, determines age of onset, with somatic instability being the rate-limiting driver of disease onset. Identified multiple DNA repair genes as genetic modifiers of disease onset. | Challenged the dogma that polyQ protein toxicity is the primary driver of onset timing, expanding drug development focus toward slowing SER, rather than solely lowering mHTT levels. |
| Ciosi *et al.* [77] | 2019 | 734 pwHD (TRACK-HD & ENROLL-HD) | Showed that somatic expansion in blood DNA predicts age of onset and disease progression, with pure CAG length—rather than polyQ length—being the best predictor of clinical decline. Genetic variants in DNA repair genes regulate somatic expansion levels in blood. | Validated somatic expansion in blood as a biomarker and provided mechanistic link that DNA repair genes modify disease severity by altering somatic instability. |
| Al Shweiki *et al*. [78] | 2020 | 16 pwHD,  17 sALS,  26 AD/MCI,  16 FTD,  11 PD,  55 controls | While CSF NfL was elevated in HD, ALS, AD and FTD, it could not distinguish between them; CSF PDYN peptides, however, were decreased only in HD. | Established CSF PDYN as a biomarker for HD, reflecting the state of MSNs. |
| Niemelä *et al.* [79] | 2021 | 13 pre-CMD,  12 post-CMD,  38 controls | Unbiased screening identified PENK as the top downregulated protein in the CSF pwHD, correlating strongly with clinical severity. | Established CSF PENK as a biomarker for HD, reflecting the state of MSNs. |

**Abbreviations:** NfL, neurofilament light; mHTT, mutant huntingtin; PENK, proenkephalin; PDYN, prodynorphin; SER, somatic expansion rate; pre-CMD, before clinical motor diagnosis of HD; post-CMD, after clinical motor diagnosis of HD; pwHD, persons with Huntington’s disease; CSF, cerebrospinal fluid; MRI, magnetic resonance imaging; HD, Huntington’s disease; ASO, antisense oligonucleotide; EHDN, European Huntington’s Disease Network; CAG, cytosine-adenine-guanine; polyQ, polyglutamine; DNA, deoxyribonucleic acid; sALS, sporadic amyotrophic lateral sclerosis; AD, Alzheimer’s disease; MCI, mild cognitive impairment; FTD, frontotemporal dementia; PD, Parkinson’s disease; MSNs, medium spiny neurons.

**Supplementary Table 5**. Key historical evidence of the structural imaging biomarkers in Huntington’s disease prior to 1st January 2022. Evidence is limited to human studies.

| **PREDICT-HD** | | | | |
| --- | --- | --- | --- | --- |
| **First author** | **Year** | **N** | **Key findings** | **Historical significance** |
| Paulsen *et al.* [80,81] | 2008 | 438 pre-CMD | Striatal atrophy detectable up to 20 years before motor onset; strong correlation with estimated years to diagnosis | Established long phase before clinical motor diagnosis; first large natural‑history cohort with imaging data |
|  | 2014 | 1,078 pre-CMD | Baseline MRI improves prediction of motor conversion | Linked structural imaging to phenoconversion trajectory |
| Aylward *et al.* [82] | 2011 | 211 pre-CMD, 60 controls | Progressive striatal and cortical atrophy with accelerated atrophy closer to onset | Defined longitudinal volumetric trajectories used in trial modelling |
| **TRACK-HD** | | | | |
| **First author** | **Year** | **N** | **Key findings** | **Historical significance** |
| Tabrizi *et al.* [83–85] | 2009 | 120 pre-CMD, 123 early post-CMD, 123 controls | Graded cross‑sectional atrophy across pre- to early post-CMD | Set multi‑site imaging standards, technically validating 3T MRI endpoints |
|  | 2011 | 116 pre-CMD, 114 early post-CMD, 115 controls | Robust annual caudate and putamen atrophy in pre-CMD, exceeding clinical change | Showed MRI more sensitive than clinical scores after 12 months |
|  | 2012 | 117 pre-CMD, 116 early post-CMD, 116 controls | Progressive GM/WM degeneration; continued divergence in pre-CMD | Strengthened use of MRI progression markers for trials |
| Stout *et al.* [86] | 2012 | 117 pre-CMD, 116 early post-CMD, 116 controls | Links between caudate/cortical loss and cognitive decline | Clarified structure–function relationships during pre-CMD phase |
| **IMAGE-HD** | | | | |
| **First author** | **Year** | **N** | **Key findings** | **Historical significance** |
| Domínguez *et al.* [87] | 2013 | 31 pre-CMD, 31 early post-CMD, 29 controls | Longitudinal caudate volume change was the only measure able to stratify participants into far from diagnosis (>15 years), close to diagnosis (<15 years) and after diagnosis groups | Confirmed caudate atrophy as one of the most sensitive early biomarkers of neurodegeneration onset in HD |
| **HD Young Adult Study (HD-YAS)** | | | | |
| **First author** | **Year** | **N** | **Key findings** | **Historical significance** |
| Scahill *et al.* [74] | 2020 | 64 pre-CMD, 67 controls | Minimal macrostructural atrophy and subtle WM alterations decades before onset | Defined earliest detectable disease-related changes |
| **Other Cohorts** | | | | |
| **First author** | **Year** | **N** | **Key findings** | **Historical significance** |
| Rosas *et al.* [88] | 2002 | 11 post-CMD, 13 controls | Early cortical thinning beyond striatum, including parietal and occipital involvement | First evidence of widespread cortical pathology in HD |

**Abbreviations:** pre-CMD, before clinical motor diagnosis of HD; sMRI, structural magnetic resonance imaging; post-CMD, after clinical motor diagnosis of HD; 3T, 3 Tesla; GM = grey matter; WM, white matter.

Interventional Trials in Huntington’s Disease

**Supplementary Table 6**. Clinical intervention trials in Huntington’s disease evaluating the therapies discussed in this review.

| **Intervention** | **Sponsor** | **Trial name** | **Trial No.** | **Phase** | **Endpoints** | **Status (2025)** | **Notes** |
| --- | --- | --- | --- | --- | --- | --- | --- |
| **SKY-0515**  (splicing modulator; dual HTT + PMS1) | Skyhawk Therapeutics | FALCON-HD | NCT06873334 | II/III | Primary: safety/tolerability;  Secondary: blood mHTT, volumetric MRI, cUHDRS | Recruiting | Targets HTT and DDR gene PMS1; tests CAG-stabilising hypothesis |
| **Tominersen**  (ASO, non-allele-selective) | Roche/Ionis | GENERATION-HD2 | NCT05686551 | II | Primary: safety/pharmacokinetics; Secondary: CSF mHTT, MRI volumetry, plasma NfL | Active | Lower-dose, early-stage cohort; designed after HD1 post-hoc signals |
| **Votoplam** (formerly **PTC518**;  (small-molecule splicing modulator) | PTC Therapeutics/Novartis | PIVOT-HD | NCT05358717 | II | Primary: safety/pharmacokinetics; Secondary: CSF mHTT, CSF NfL, MRI | Active | Oral therapy; dose-dependent mHTT lowering; 24-month follow-up |
| **AMT-130**  (AAV5-mHTT gene therapy) | uniQure | US study  EU study | NCT04120493  NCT05243017 | I/II  Ib/II | Primary: safety;  Secondary: CSF NfL, cUHDRS  Safety and exploratory biomarker endpoints | Active  Active | 36-month interim: 75% slower functional decline and NfL lowered below baseline  Harmonised with US protocol for pooled analysis |
| **AB-1001**  (AAV-based HTT-lowering gene therapy) | Asklepios BioPharma/Bayer | — | NCT05541627 | I/II | Primary: safety (MRI monitoring);  Exploratory: CSF NfL, CSF HTT aggregates, motor function | Active | Intrathecal delivery; dose-escalation design |
| **WVE-003**  (allele-selective ASO) | Wave Life Sciences | SELECT-HD | NCT05032196 | Ib/IIa | Primary: safety/tolerability; Secondary: CSF mHTT, CSF NfL, MRI (caudate atrophy) | Active | 20–30 % mHTT reduction up to 90 days; preserves wtHTT |
| **Tominersen**  (ASO, non-allele-selective) | Roche/Ionis | GENERATION-HD1 | NCT03761849 | III | Primary: cUHDRS efficacy/safety; Secondary: CSF mHTT, MRI | Terminated (2021) | Ventricular expansion and NfL elevated at high dose; post-hoc sub-analyses guiding HD2 |
| **Branaplam**  (small-molecule splicing modulator) | Novartis | VIBRANT-HD | NCT05111249 | IIb | Primary: safety/tolerability; Exploratory: mHTT, NfL | Terminated (2022) | Peripheral neuropathy/NfL spikes led to halt |

**Abbreviations:** HTT = huntingtin; PMS1 = postmeiotic segregation increased 1; SER = somatic expansion ratio; mHTT = mutant huntingtin; DDR = DNA damage response; CAG = cytosine-adenine-guanine; ASO = antisense oligonucleotide; CSF = cerebrospinal fluid; MRI = magnetic resonance imaging; NfL = neurofilament light; AAV5 = adeno-associated virus serotype 5; US = United States; EU = Europe; cUHDRS = composite Unified Huntington’s Disease Rating Scale; AAV = adeno-associated virus; wtHTT = wild-type huntingtin.

References

1. Ouzzani M, Hammady H, Fedorowicz Z, Elmagarmid A. Rayyan—a web and mobile app for systematic reviews. Syst Rev. 2016;5:210. <https://doi.org/10.1186/s13643-016-0384-4>

2. Sampedro F, Martínez-Horta S, Pérez-Pérez J, Pérez-González R, Horta-Barba A, Campolongo A, et al. Plasma TDP-43 Reflects Cortical Neurodegeneration and Correlates with Neuropsychiatric Symptoms in Huntington’s Disease. Clin Neuroradiol. 2022;32:1077–85. <https://doi.org/10.1007/S00062-022-01150-5>

3. Parkin GM, Corey-Bloom J, Long JD, Snell C, Smith H, Thomas EA. Associations between prognostic index scores and plasma neurofilament light in Huntington’s disease. Parkinsonism Relat Disord. 2022;97:25–8. <https://doi.org/10.1016/j.parkreldis.2022.02.023>

4. Byrne LM, Schultz JL, Rodrigues FB, van der Plas E, Langbehn D, Nopoulos PC, et al. Neurofilament Light Protein as a Potential Blood Biomarker for Huntington’s Disease in Children. Mov Disord. 2022;37:1526–31. <https://doi.org/10.1002/MDS.29027>

5. Hellem MNN, Cheong RY, Tonetto S, Vinther-Jensen T, Hendel RK, Larsen IU, et al. Decreased CSF oxytocin relates to measures of social cognitive impairment in Huntington’s disease patients. Parkinsonism Relat Disord. 2022;99:23–9. <https://doi.org/10.1016/j.parkreldis.2022.05.003>

6. Barschke P, Abu-Rumeileh S, Al Shweiki MHDR, Barba L, Paolini Paoletti F, Oeckl P, et al. Cerebrospinal fluid levels of proenkephalin and prodynorphin are differentially altered in Huntington’s and Parkinson’s disease. J Neurol. 2022;269:5136–43. <https://doi.org/10.1007/S00415-022-11187-8>

7. Fodale V, Pintauro R, Daldin M, Spiezia MC, MacDonald D, Bresciani A. Quantifying Huntingtin Protein in Human Cerebrospinal Fluid Using a Novel Polyglutamine Length-Independent Assay. J Huntingtons Dis. 2022;11:291–305. <https://doi.org/10.3233/JHD-220527>

8. Ananbeh H, Novak J, Juhas S, Juhasova J, Klempir J, Doleckova K, et al. Huntingtin Co-Isolates with Small Extracellular Vesicles from Blood Plasma of TgHD and KI-HD Pig Models of Huntington’s Disease and Human Blood Plasma. Int J Mol Sci. 2022;23:5598. <https://doi.org/10.3390/IJMS23105598/S1>

9. Huseby CJ, Delvaux E, Brokaw DL, Coleman PD. Blood Transcript Biomarkers Selected by Machine Learning Algorithm Classify Neurodegenerative Diseases including Alzheimer’s Disease. Biomolecules. 2022;12:1592. <https://doi.org/10.3390/biom12111592>

10. Caron NS, Haqqani AS, Sandhu A, Aly AE, Findlay Black H, Bone JN, et al. Cerebrospinal fluid biomarkers for assessing Huntington disease onset and severity. Brain Commun. 2022;4. <https://doi.org/10.1093/BRAINCOMMS/FCAC309>

11. Anderson KE, Stevenson M, Varghese R, Hebron ML, Koppel E, McCartin M, et al. Alteration of Autophagy and Glial Activity in Nilotinib-Treated Huntington’s Disease Patients. Metabolites. 2022;12. <https://doi.org/10.3390/METABO12121225>

12. Parkin GM, Corey-Bloom J, Snell C, Smith H, Laurenza A, Daldin M, et al. Salivary Huntingtin protein is uniquely associated with clinical features of Huntington’s disease. Sci Rep. 2023;13:1–9. <https://doi.org/10.1038/S41598-023-28019-Y;SUBJMETA>

13. Chang K-H, Cheng M-L, Lo C-J, Fan C-M, Wu Y-R, Chen C-M, et al. Alternations of Lipoprotein Profiles in the Plasma as Biomarkers of Huntington’s Disease. Cells. 2023;12. <https://doi.org/10.3390/CELLS12030385>

14. Lin TW, Chang JK, Wu YR, Sun TH, Cheng YY, Ren CT, et al. Ganglioside-focused Glycan Array Reveals Abnormal Anti-GD1b Auto-antibody in Plasma of Preclinical Huntington’s Disease. Mol Neurobiol. 2023;60:3873–82. <https://doi.org/10.1007/S12035-023-03307-W>

15. Li XY, Bao YF, Xie JJ, Gao B, Qian SX, Dong Y, et al. Application Value of Serum Neurofilament Light Protein for Disease Staging in Huntington’s Disease. Mov Disord. 2023;38:1307–15. <https://doi.org/10.1002/MDS.29430>

16. Parkin GM, Thomas EA, Corey-Bloom J. Plasma NfL as a prognostic biomarker for enriching HD-ISS stage 1 categorisation: a cross-sectional study. EBioMedicine. 2023;93. <https://doi.org/10.1016/j.ebiom.2023.104646>

17. Hwang YS, Oh E, Kim M, Lee CY, Kim HS, Chung SJ, et al. Plasma neurofilament light-chain and phosphorylated tau as biomarkers of disease severity in Huntington’s disease: Korean cohort data. J Neurol Sci. 2023;452:120744. <https://doi.org/10.1016/J.JNS.2023.120744>

18. Gijs M, Jorna N, Datson N, Beekman C, Dansokho C, Weiss A, et al. High Levels of Mutant Huntingtin Protein in Tear Fluid From Huntington’s Disease Gene Expansion Carriers. J Mov Disord. 2024;17:181–8. <https://doi.org/10.14802/JMD.24014>

19. Turner M, Bartlett D, Poudel G, Zaenker P, Laws S, Lo J, et al. Associations between Sleep Quality and Serum Levels of Neurofilament Light in Individuals with Premanifest Huntington Disease. Sleep Science. 2024;17:e199–202. <https://doi.org/10.1055/S-0043-1777783>

20. Cao LX, Yin JH, Du G, Yang Q, Huang Y. Identifying and verifying Huntington’s disease subtypes: Clinical features, neuroimaging, and cytokine changes. Brain Behav. 2024;14:e3469. <https://doi.org/10.1002/BRB3.3469;WGROUP:STRING:PUBLICATION>

21. Martinez-Horta S, Perez-Perez J, Perez-Gonzalez R, Sampedro F, Horta-Barba A, Campolongo A, et al. Cognitive phenotype and neurodegeneration associated with Tau in Huntington’s disease. Ann Clin Transl Neurol. 2024;11:1160–71. <https://doi.org/10.1002/ACN3.52031>

22. Korpela S, Sundblom J, Zetterberg H, Constantinescu R, Svenningsson P, Paucar M, et al. Cerebrospinal fluid glial fibrillary acidic protein, in contrast to amyloid beta protein, is associated with disease symptoms in Huntington’s disease. J Neurol Sci. 2024;459. <https://doi.org/10.1016/j.jns.2024.122979>

23. Parkin GM, Thomas EA, Corey-Bloom J. Mapping neurodegeneration across the Huntington’s disease spectrum: a five-year longitudinal analysis of plasma neurofilament light. EBioMedicine. 2024;104:105173. <https://doi.org/10.1016/J.EBIOM.2024.105173>

24. Machacek M, Garcia-Montoya E, McColgan P, Sanwald-Ducray P, Mazer NA. NfL concentration in CSF is a quantitative marker of the rate of neurodegeneration in aging and Huntington’s disease: a semi-mechanistic model-based analysis. Front Neurosci. 2024;18. <https://doi.org/10.3389/FNINS.2024.1420198>

25. Chiang PI, Chang KH, Tang HY, Wu YR, Cheng ML, Chen CM. Diagnostic Potential of Alternations of Bile Acid Profiles in the Plasma of Patients with Huntington’s Disease. Metabolites. 2024;14. <https://doi.org/10.3390/METABO14070394>

26. Voysey ZJ, Owen NE, Holbrook JA, Malpetti M, Le Draoulec C, Spindler LRB, et al. A 14-year longitudinal study of neurofilament light chain dynamics in premanifest and transitional Huntington’s disease. Journal of Neurology. 2024;271:7572–82. <https://doi.org/10.1007/S00415-024-12700-X>

27. Herrero-Lorenzo M, Pérez-Pérez J, Escaramís G, Martínez-Horta S, Pérez-González R, Rivas-Asensio E, et al. Small RNAs in plasma extracellular vesicles define biomarkers of premanifest changes in Huntington’s disease. J Extracell Vesicles. 2024;13. <https://doi.org/10.1002/JEV2.12522>

28. Anderson DG, Ferreira-Correia A, Rodrigues FB, Byrne LM, Wild EJ, Krause A. Comparative analysis of neurofilament light chain in Huntington’s disease like 2 and Huntington’s disease. J Huntingtons Dis. 2025;14:103–8. <https://doi.org/10.1177/18796397241300141>

29. Voysey ZJ, Goodman AOG, Rogers L, Holbrook JA, Lazar AS, Barker RA. Sleep abnormalities are associated with greater cognitive deficits and disease activity in Huntington’s disease: a 12-year polysomnographic study. Brain Commun. 2025;7. <https://doi.org/10.1093/BRAINCOMMS/FCAF126>

30. Bamford AR, Parkin GM, Corey-Bloom J, Thomas EA. Comparisons of neurodegenerative disease biomarkers across different biological fluids from patients with Huntington’s disease. J Neurol. 2025;272:1–12. <https://doi.org/10.1007/S00415-024-12785-4/FIGURES/4>

31. McGarry A, Moaddel R. A Pilot Proteomic Analysis of Huntington’s Disease by Functional Capacity. Brain Sci. 2025;15. <https://doi.org/10.3390/BRAINSCI15010076/S1>

32. Scahill RI, Farag M, Murphy MJ, Hobbs NZ, Leocadi M, Langley C, et al. Somatic CAG repeat expansion in blood associates with biomarkers of neurodegeneration in Huntington’s disease decades before clinical motor diagnosis. Nat Med. 2025;31:807–18. <https://doi.org/10.1038/S41591-024-03424-6>

33. Heim B, Mandler E, Peball M, Carbone F, Schwarzová K, Demjaha R, et al. Serum neurofilament light chain but not serum glial fibrillary acidic protein is a marker of early Huntington’s disease. J Neurol. 2025;272. <https://doi.org/10.1007/S00415-025-12901-Y>

34. Alpaugh M, Lantero-Rodriguez J, Benedet AL, Manseau U, Boutin M, Maiuri M, et al. Tau levels in platelets isolated from Huntington’s disease patients serve as a biomarker of disease severity. J Neurol. 2025;272. <https://doi.org/10.1007/S00415-025-12966-9>

35. Leckey CA, Coulton JB, Giovannucci TA, He Y, Aslanyan A, Laban R, et al. CSF neurofilament light chain profiling and quantitation in neurological diseases. Brain Commun. 2024;6. <https://doi.org/10.1093/braincomms/fcae132>

36. Owen NE, Voysey ZJ, Holbrook JA, Malpetti M, Le Draoulec C, Spindler LRB, et al. A 14-Year Study of Serum Glial Fibrillary Acidic Protein and Total Tau in Premanifest Huntington’s. Ann Clin Transl Neurol. 2025;12:1296–301. <https://doi.org/10.1002/ACN3.70057>

37. Qian SX, Bao YF, Li XY, Dong Y, Zhang XL, Wu ZY. Multi-omics Analysis Reveals Key Gut Microbiota and Metabolites Closely Associated with Huntington’s Disease. Mol Neurobiol. 2025;62:351–65. <https://doi.org/10.1007/S12035-024-04271-9>

38. Khetarpal V, Herbst T, Dominguez C, Munoz-Sanjuan I, Sampaio C, Marks B, et al. Lack of Evidence for Kynurenine Pathway Dysfunction in Huntington’s Disease: CSF and Plasma Analyses from the HDClarity Study. J Huntingtons Dis. 2025;14:85–92. <https://doi.org/10.1177/18796397241301761>

39. Estevez-Fraga C, Sebenius I, Hansen JY, Hänisch B, Zeun P, Scahill RI, et al. Cell-specific mechanisms drive connectivity across the time course of Huntington’s disease. Nature Communications. 2025;16:5519. <https://doi.org/10.1038/s41467-025-60556-0>

40. Coleman A, Touzé A, Farag M, Pengo M, Murphy MJ, Hassan Y, et al. Evaluating finger-prick blood collection for remote quantification of neurofilament light in neurological diseases. J Neurol. 2025;272. <https://doi.org/10.1007/S00415-025-13232-8>

41. Farag M, Murphy MJ, Hobbs NZ, Leocadi M, Fayer K, Thackeray O, et al. Cerebrospinal Fluid Proenkephalin Predicts Striatal Atrophy Decades before Clinical Motor Diagnosis in Huntington’s Disease. Movement Disorders. 2025. <https://doi.org/10.1002/mds.70062>

42. Meda FJ, Dittrich A, Skoog I, Kern S, Leckey C, Wild EJ, et al. Alpha-internexin as a brain-specific neurodegeneration marker: development and validation of a novel CSF assay. Journal of Neurology. 2025;272:676. <https://doi.org/10.1007/S00415-025-13428-Y>

43. Zeun P, McColgan P, Dhollander T, Gregory S, Johnson EB, Papoutsi M, et al. Timing of selective basal ganglia white matter loss in premanifest Huntington’s disease. Neuroimage Clin. 2022;33:102927. <https://doi.org/10.1016/J.NICL.2021.102927>

44. Barrios-Martinez J V., Fernandes-Cabral DT, Abhinav K, Fernandez-Miranda JC, Chang YF, Suski V, et al. Differential tractography as a dynamic imaging biomarker: A methodological pilot study for Huntington’s disease. Neuroimage Clin. 2022;35:103062. <https://doi.org/10.1016/J.NICL.2022.103062>

45. Lavrador R, Júlio F, Januário C, Castelo-Branco M, Caetano G. Classification of Huntington’s Disease Stage with Features Derived from Structural and Diffusion-Weighted Imaging. J Pers Med. 2022;12:704. <https://doi.org/10.3390/jpm12050704>

46. Schmid RD, Remlinger J, Abegg M, Hoepner R, Hoffmann R, Lukas C, et al. No optical coherence tomography changes in premanifest Huntington’s disease mutation carriers far from disease onset. Brain Behav. 2022;12. <https://doi.org/10.1002/brb3.2592>

47. Tan B, Shishegar R, Fornito A, Poudel G, Georgiou-Karistianis N. Longitudinal mapping of cortical surface changes in Huntington’s Disease. Brain Imaging Behav. 2022;16:1381–91. <https://doi.org/10.1007/s11682-021-00625-2>

48. Casella C, Chamberland M, Laguna PL, Parker GD, Rosser AE, Coulthard E, et al. Mutation‐related magnetization‐transfer, not axon density, drives white matter differences in premanifest Huntington disease: Evidence from in vivo ultra‐strong gradient MRI Hum Brain Mapp. 2022;43:3439–60. <https://doi.org/10.1002/hbm.25859>

49. Tan B, Shishegar R, Oldham S, Fornito A, Poudel G, Georgiou-Karistianis N. Investigating longitudinal changes to frontal cortico-striatal tracts in Huntington’s disease: the IMAGE-HD study. Brain Imaging Behav. 2022;16:2457–66. <https://doi.org/10.1007/s11682-022-00699-6>

50. Ponomareva N V., Klyushnikov SA, Abramycheva N, Konovalov RN, Krotenkova M, Kolesnikova E, et al. Neurophysiological hallmarks of Huntington’s disease progression: an EEG and fMRI connectivity study. Front Aging Neurosci. 2023;15. <https://doi.org/10.3389/fnagi.2023.1270226>

51. Yao J, Morrison MA, Jakary A, Avadiappan S, Chen Y, Luitjens J, et al. Comparison of quantitative susceptibility mapping methods for iron-sensitive susceptibility imaging at 7T: An evaluation in healthy subjects and patients with Huntington’s disease. Neuroimage. 2023;265:119788. <https://doi.org/10.1016/j.neuroimage.2022.119788>

52. Dusek P, Kopal A, Brichova M, Roth J, Ulmanova O, Klempir J, et al. Is retina affected in Huntington’s disease? Is optical coherence tomography a good biomarker? PLoS One. 2023;18:e0282175. <https://doi.org/10.1371/journal.pone.0282175>

53. Hu B, Younes L, Bu X, Liu C-F, Ratnanather JT, Paulsen J, et al. Mixed longitudinal and cross-sectional analyses of deep gray matter and white matter using diffusion weighted images in premanifest and manifest Huntington’s disease. Neuroimage Clin. 2023;39:103493. <https://doi.org/10.1016/j.nicl.2023.103493>

54. Lemoine L, Lunven M, Fraisse N, Youssov K, Bapst B, Morgado G, et al. The striatum in time production: The model of Huntington’s disease in longitudinal study. Neuropsychologia. 2023;179:108459. <https://doi.org/10.1016/J.NEUROPSYCHOLOGIA.2022.108459>

55. Delva A, Van Laere K, Vandenberghe W. Longitudinal Imaging of Regional Brain Volumes, SV2A, and Glucose Metabolism In Huntington’s Disease. Movement Disorders. 2023;38:1515–26. <https://doi.org/10.1002/mds.29501>

56. Stoebner ZA, Hett K, Lyu I, Johnson H, Paulsen JS, Long JD, et al. Comprehensive shape analysis of the cortex in Huntington’s disease. Hum Brain Mapp. 2023;44:1417–31. <https://doi.org/10.1002/hbm.26125>

57. Estevez‐Fraga C, Elmalem MS, Papoutsi M, Durr A, Rees EM, Hobbs NZ, et al. Progressive alterations in white matter microstructure across the timecourse of Huntington’s disease. Brain Behav. 2023;13. <https://doi.org/10.1002/brb3.2940>

58. Wilkes FA, Jakabek D, Walterfang M, Velakoulis D, Poudel GR, Stout JC, et al. Hippocampal morphology in Huntington’s disease, implications for plasticity and pathogenesis: The IMAGE-HD study. Psychiatry Res Neuroimaging. 2023;335:111694. <https://doi.org/10.1016/J.PSCYCHRESNS.2023.111694>

59. Wilkes FA, Jakabek D, Walterfang M, Velakoulis D, Poudel GR, Stout JC, et al. The shape of things to come. Mapping spatiotemporal progression of striatal morphology in Huntington disease: The IMAGE-HD study. Psychiatry Res Neuroimaging. 2023;335:111717. <https://doi.org/10.1016/J.PSCYCHRESNS.2023.111717>

60. Demir A, Rosas HD. Altered interhemispheric connectivity in Huntington’s Disease. Neuroimage Clin. 2024;44:103670. <https://doi.org/10.1016/J.NICL.2024.103670>

61. Ghofrani-Jahromi M, Poudel GR, Razi A, Abeyasinghe PM, Paulsen JS, Tabrizi SJ, et al. Prognostic enrichment for early-stage Huntington’s disease: An explainable machine learning approach for clinical trial. Neuroimage Clin. 2024;43:103650. <https://doi.org/10.1016/J.NICL.2024.103650>

62. Yin J-H, Liu Y-O, Li H-L, Burgunder JM, Huang Y. White Matter Microstructure Changes Revealed by Diffusion Kurtosis and Diffusion Tensor Imaging in Mutant Huntingtin Gene Carriers. J Huntingtons Dis. 2024;13:301–13. <https://doi.org/10.3233/JHD-240018>

63. van Wamelen DJ, Martin NH, Makos O, Badenoch J, Valera-Bermejo JM, Hartmann M, et al. Study protocol for the iMarkHD study in individuals with Huntington’s disease. J Huntingtons Dis. 2024;13:479–89. <https://doi.org/10.1177/18796397241288165>

64. Barrett MJ, Negida A, Mukhopadhyay N, Kim JK, Nawaz H, Jose J, et al. Optimizing Screening for Intrastriatal Interventions in Huntington’s Disease Using Predictive Models. Movement Disorders. 2024;39:855–62. <https://doi.org/10.1002/mds.29749>

65. Yao J, Morrison MA, Jakary A, Avadiappan S, Rowley P, Glueck J, et al. Altered Iron and Microstructure in Huntington’s Disease Subcortical Nuclei: Insight From 7T MRI. Journal of Magnetic Resonance Imaging. 2024;60:1484–99. <https://doi.org/10.1002/jmri.29195>

66. De Paepe AE, Bikou V, Turan E, Pérez-Bellido A, Garcia-Gorro C, Rodriguez-Dechicha N, et al. Striato-cortical connectivity patterns predict clinical profiles in Huntington’s disease. Neuroimage Clin. 2025;46. <https://doi.org/10.1016/j.nicl.2025.103788>

67. Giannoula A, De Paepe AE, Sanz F, Furlong LI, Camara E. Identifying time patterns in Huntington’s disease trajectories using dynamic time warping-based clustering on multi-modal data. Sci Rep. 2025;15:3081. <https://doi.org/10.1038/s41598-025-86686-5>

68. Abeyasinghe PM, Cole JH, Razi A, Poudel GR, Paulsen JS, Tabrizi SJ, et al. Brain Age as a New Measure of Disease Stratification in Huntington’s Disease. Movement Disorders. 2025;40:627–41. <https://doi.org/10.1002/mds.30109>

69. Byrne LM, Rodrigues FB, Blennow K, Durr A, Leavitt BR, Roos RAC, et al. Neurofilament light protein in blood as a potential biomarker of neurodegeneration in Huntington’s disease: a retrospective cohort analysis. Lancet Neurol. 2017;16:601–9. <https://doi.org/10.1016/S1474-4422(17)30124-2>

70. Johnson EB, Byrne LM, Gregory S, Rodrigues FB, Blennow K, Durr A, et al. Neurofilament light protein in blood predicts regional atrophy in Huntington disease. Neurology. 2018;90:e717–23. [https://doi.org/10.1212/WNL.0000000000005005](https://doi.org/10.1212/WNL.0000000000005005;REQUESTEDJOURNAL:JOURNAL:WNL;JOURNAL:JOURNAL:WNL;WGROUP:STRING:PUBLICATION)

71. Wild EJ, Boggio R, Langbehn D, Robertson N, Haider S, Miller JRC, et al. Quantification of mutant huntingtin protein in cerebrospinal fluid from Huntington’s disease patients. J Clin Invest. American Society for Clinical Investigation; 2015;125:1979–86. <https://doi.org/10.1172/JCI80743>

72. Byrne LM, Rodrigues FB, Johnson EB, Wijeratne PA, De Vita E, Alexander DC, et al. Evaluation of mutant huntingtin and neurofilament proteins as potential markers in Huntington’s disease. Sci Transl Med. 2018;10:7108. [https://doi.org/10.1126/SCITRANSLMED.AAT7108](https://doi.org/10.1126/SCITRANSLMED.AAT7108;PAGE:STRING:ARTICLE/CHAPTER)

73. Rodrigues FB, Byrne LM, Tortelli R, Johnson EB, Wijeratne PA, Arridge M, et al. Mutant huntingtin and neurofilament light have distinct longitudinal dynamics in Huntington’s disease. Sci Transl Med. 2020;12:eabc2888. <https://doi.org/10.1126/SCITRANSLMED.ABC2888>

74. Scahill RI, Zeun P, Osborne-Crowley K, Johnson EB, Gregory S, Parker C, et al. Biological and clinical characteristics of gene carriers far from predicted onset in the Huntington’s disease Young Adult Study (HD-YAS): a cross-sectional analysis. Lancet Neurol. 2020;19:502. <https://doi.org/10.1016/S1474-4422(20)30143-5>

75. Tabrizi SJ, Leavitt BR, Landwehrmeyer GB, Wild EJ, Saft C, Barker RA, et al. Targeting Huntingtin Expression in Patients with Huntington’s Disease. New England Journal of Medicine. 2019;380:2307–16. <https://doi.org/10.1056/NEJMOA1900907;ISSUE:ISSUE:DOI>

76. Lee JM, Correia K, Loupe J, Kim KH, Barker D, Hong EP, et al. CAG Repeat Not Polyglutamine Length Determines Timing of Huntington’s Disease Onset. Cell. 2019;178:887-900.e14. <https://doi.org/10.1016/j.cell.2019.06.036>

77. Ciosi M, Maxwell A, Cumming SA, Hensman Moss DJ, Alshammari AM, Flower MD, et al. A genetic association study of glutamine-encoding DNA sequence structures, somatic CAG expansion, and DNA repair gene variants, with Huntington disease clinical outcomes. EBioMedicine. 2019;48:568–80. <https://doi.org/10.1016/j.ebiom.2019.09.020>

78. Al Shweiki MHDR, Oeckl P, Pachollek A, Steinacker P, Barschke P, Halbgebauer S, et al. Cerebrospinal Fluid Levels of Prodynorphin-Derived Peptides are Decreased in Huntington’s Disease. Movement Disorders. 2021;36:492–7. [https://doi.org/10.1002/MDS.28300](https://doi.org/10.1002/MDS.28300;WGROUP:STRING:PUBLICATION)

79. Niemela V, Landtblom AM, Nyholm D, Kneider M, Constantinescu R, Paucar M, et al. Proenkephalin Decreases in Cerebrospinal Fluid with Symptom Progression of Huntington’s Disease. Movement Disorders. 2021;36:481–91. [https://doi.org/10.1002/MDS.28391](https://doi.org/10.1002/MDS.28391;PAGE:STRING:ARTICLE/CHAPTER)

80. Paulsen JS, Langbehn DR, Stout JC, Aylward E, Ross CA, Nance M, et al. Detection of Huntington’s disease decades before diagnosis: the Predict-HD study. J Neurol Neurosurg Psychiatry. 2008;79:874–80. <https://doi.org/10.1136/jnnp.2007.128728>

81. Paulsen JS, Long JD, Ross CA, Harrington DL, Erwin CJ, Williams JK, et al. Prediction of manifest Huntington’s disease with clinical and imaging measures: a prospective observational study. Lancet Neurol. 2014;13:1193–201. <https://doi.org/10.1016/S1474-4422(14)70238-8>

82. Aylward EH, Nopoulos PC, Ross CA, Langbehn DR, Pierson RK, Mills JA, et al. Longitudinal change in regional brain volumes in prodromal Huntington disease. J Neurol Neurosurg Psychiatry. 2011;82:405–10. <https://doi.org/10.1136/jnnp.2010.208264>

83. Tabrizi SJ, Langbehn DR, Leavitt BR, Roos RA, Durr A, Craufurd D, et al. Biological and clinical manifestations of Huntington’s disease in the longitudinal TRACK-HD study: cross-sectional analysis of baseline data. Lancet Neurol. 2009;8:791–801. <https://doi.org/10.1016/S1474-4422(09)70170-X>

84. Tabrizi SJ, Scahill RI, Durr A, Roos RA, Leavitt BR, Jones R, et al. Biological and clinical changes in premanifest and early stage Huntington’s disease in the TRACK-HD study: the 12-month longitudinal analysis. Lancet Neurol. 2011;10:31–42. <https://doi.org/10.1016/S1474-4422(10)70276-3>

85. Tabrizi SJ, Reilmann R, Roos RA, Durr A, Leavitt B, Owen G, et al. Potential endpoints for clinical trials in premanifest and early Huntington’s disease in the TRACK-HD study: analysis of 24 month observational data. Lancet Neurol. 2012;11:42–53. <https://doi.org/10.1016/S1474-4422(11)70263-0>

86. Stout JC, Jones R, Labuschagne I, O’Regan AM, Say MJ, Dumas EM, et al. Evaluation of longitudinal 12 and 24 month cognitive outcomes in premanifest and early Huntington’s disease. J Neurol Neurosurg Psychiatry. 2012;83:687–94. <https://doi.org/10.1136/jnnp-2011-301940>

87. Domínguez D JF, Egan GF, Gray MA, Poudel GR, Churchyard A, Chua P, et al. Multi-Modal Neuroimaging in Premanifest and Early Huntington’s Disease: 18 Month Longitudinal Data from the IMAGE-HD Study. PLoS One. 2013;8:e74131. <https://doi.org/10.1371/journal.pone.0074131>

88. Rosas HD, Liu AK, Hersch S, Glessner M, Ferrante RJ, Salat DH, et al. Regional and progressive thinning of the cortical ribbon in Huntington’s disease. Neurology. 2002;58:695–701. <https://doi.org/10.1212/WNL.58.5.695>
